# Discovery of novel hammerhead, twister, and DVRz-associated circular RNAs in *Vitaceae*, *Solanaceae,* and *Rosaceae*

**DOI:** 10.1128/msystems.01168-25

**Published:** 2025-09-15

**Authors:** Ali Raza, Yu Zhu, Ming'an Deng, Qingfa Wu

**Affiliations:** 1 Department of Pharmacy, The First Affiliated Hospital of USTC, Division of Life Sciences and Medicine, University of Science and Technology of China 612146 , Hefei, Anhui, China; 2 Key Laboratory of Anhui Province for Emerging and Reemerging Infectious Diseases, University of Science and Technology of China 12652, Hefei, Anhui, China; University of Massachusetts Amherst, Amherst, Massachusetts, USA

**Keywords:** viroid, circular RNA, ribozyme, twister viroid-like RNA, ambi-like virus, Obelisk-like RNA

## Abstract

Viroids and viroid-like exogenous circular RNAs (ex-circRNAs) are small infectious RNA agents capable of autonomous replication within host cells, independent of any helper virus. In this study, we analyzed rRNA-depleted transcriptomic data sets from members of the *Vitaceae*, *Solanaceae*, and *Rosaceae* families to explore the diversity of these subviral elements. Using both homology-based and alignment-free ribozyme detection methods, we recovered 16 known viroids and identified 13 novel viroid-like RNAs from plant metatranscriptomic data sets. Among these, we report two new hammerhead ribozyme-containing viroid-like RNAs—CGHVd-RNA in *Physalis peruviana* and GPLMVd-RNA in *Vitis vinifera*—that display distinct secondary structures and limited sequence similarity to known viroids, supporting their classification as new species within the *Avsunviroidae* family. We also identified a symmetric twister ribozyme-containing ex-circRNA in peach, with validated *in vitro* self-cleavage activity. Additionally, three ambi-like circular RNAs were detected in grapevine and peach, encoding divergent RNA-dependent RNA polymerases, hairpin (HP), and delta virus ribozymes (DVRz). Finally, a novel Obelisk-like RNA with unique structural features was discovered in the Cape gooseberry. These results reveal the hidden complexity and broad host range of plant-associated viroid-like RNAs and suggest the existence of previously unrecognized subviral lineages with distinct evolutionary origins.

**IMPORTANCE:**

This study reveals remarkable diversity in plant-associated subviral RNA populations, identifying 13 novel viroid-like RNAs alongside 16 known viroids. Notable discoveries include new hammerhead ribozyme-containing species-level ex-circRNAs from Cape gooseberry and grapevine, a symmetric twister ribozyme ex-circRNA in peach, and, surprisingly, ambi-like circular RNAs with RNA-dependent RNA polymerases. These findings significantly expand our understanding of the complexity and evolutionary diversity of plant-associated subviral RNAs.

## INTRODUCTION

Viroids are RNA molecules, which are small (245–400 nucleotides), single-stranded, circular, non-encapsidated, and non-protein-coding. They possess high levels of self-complementary sequences which result in the formation of secondary structures. Viroids are classified into two families based on structural features and functionality: the *Pospiviroidae* and the *Avsunviroidae* (1–3). Viroids of the *Pospiviroidae* family contain a central conserved region (CCR) within a rod-like secondary structure (2) and replicate in the infected host cell’s nucleus (4). In contrast, viroids of the *Avsunviroidae* family do not possess a CCR, adopt a secondary branch-like structure, contain hammerhead ribozymes, and replicate in the host cell’s plastids (2, 5–7). Members of the family *Avsunviroidae* infect only dicotyledonous plants, including both herbaceous and woody species (2). In contrast, members of the family Pospiviroidae can infect both monocotyledonous and dicotyledonous plants (8). Recent data show that viroids affect nearly 35 plant families, causing significant damage to crops. The three families most affected by viroids and their variants are *Rutaceae*, *Solanaceae,* and *Rosaceae* (9).

Grapevine, belonging to the *Vitaceae* family, represents one of the world’s most economically important fruit crops, with significant cultural and historical value in many regions. Seven species of viroids are known to infect grapevines, namely *Hop stunt viroid* (HSVd) (10, 11), *Citrus exocortis viroid* (CEVd) (12), *Australian grapevine viroid* (AGVd) (13), *Grapevine yellow speckle viroid 1* (GYSVd-1) (14), *Grapevine yellow speckle viroid 2* (GYSVd-2) (15), *Grapevine latent viroid* (GLVd) (16), and *Japanese grapevine viroid* (17, 18). A hammerhead viroid-like RNA (GHVd) was detected in grapevines in California, Italy, France, and Greece; however, its true viroid nature has yet to be determined and is regarded as a putative viroid species (19–22). GYSVd-1 and GYSVd-2 are the causal agents of yellow speckle disease, which is characterized by yellow spots on the grapevine leaves (15). These two viroids also exhibit a synergism with grapevine fanleaf virus, which results in severe vein banding in grapevine (23). The remaining viroid species do not result in disease symptoms in grapevines (24), although CEVd and HSVd do cause disease symptoms in other host plants. Both GYSVd-1 and GYSVd-2 have a global distribution and are found in nearly all grape growing regions (24), although GYSVd-1 has a greater level of diversity when compared with GYSVd-2 (25, 26). AGVd is one of the least studied of the grapevine viroids; however, population studies suggest that it occurs as a series of variants clustering into three possible groups based on geographical origin and introduction route (27–30). CEVd occurs as a range of variants falling in two groups with no association to host or geographical origin (31). Population studies on HSVd have demonstrated a series of variants which often cluster together based on host origin, with grapevine isolates clustering together (31, 32). Phylogenetic analyses have suggested that HSVd originated in grapevine and “jumped” into hops, resulting in hop stunt disease. Sano et al. (32) have also suggested that HSVd in hops is in the process of becoming adapted to this new host and is therefore virulent in hops. GLVd was first reported from symptomless grapevines in China (16) and Italy (33).

Family *Solanaceae*, commonly known as nightshades, has historically been the most extensively studied plant family for viroid infections. *Potato spindle tuber viroid* (PSTVd), the type species of the genus Pospiviroid within the family *Pospiviroidae*, was the first viroid discovered and remains one of the most economically significant and extensively studied viroids (34, 35). *Tomato planta macho viroid* (TPMVd), *Tomato apical stunt viroid* (TASVd), *Columnea latent viroid* (CLVd), and *Mexican papita viroid* (MPVd) represent additional members of the genus Pospiviroid that infect solanaceous hosts (1, 8, 36, 37). These viroids share considerable sequence homology with PSTVd and often produce similar symptomatology, complicating accurate diagnosis without molecular techniques (38). *Chrysanthemum stunt viroid* (CSVd), although primarily associated with ornamental plants, has also been reported in solanaceous hosts, demonstrating the complex host range dynamics of many viroids (39). *Tomato chlorotic dwarf viroid* (TCDVd), first identified in Canada in tomato plants displaying stunt growth and leaf chlorosis, represents a relatively more recent addition to the list of viroids infecting *Solanaceae* family (40). Molecular analysis revealed its close relationship to PSTVd, with approximately 90% sequence homology; however, it maintains a distinct pathogenicity profile (8, 41). Notably, *Citrus exocortis viroid* (CEVd), despite its name suggesting citrus as the primary host, naturally infects several solanaceous species and can cause significant damage to tomato crops (42, 43). This underscores the complex and often overlapping host ranges that characterize many viroid species. Additionally, *Eggplant latent viroid* (ELVd) represents a unique case within solanaceous viroids. Unlike most members of the *Pospiviroidae* family, ELVd infections are typically latent or asymptomatic in eggplant (44, 45). This latency has important implications for viroid epidemiology, as asymptomatic hosts can serve as reservoirs for viral particles, facilitating transmission to more susceptible crops (46, 47). The molecular mechanisms underlying pathogenicity in solanaceous viroids have been the subject of intensive investigation (48, 49).

The *Rosaceae* family encompasses numerous economically important fruit crops, including apple, pear, peach, plum, cherry, and strawberry. Several viroids have been identified in *Rosaceae* members, with *Apple scar skin viroid* (ASSVd) and *Pear blister canker viroid* (PBCVd) being the most extensively studied (46). ASSVd, the type species of the genus Apscaviroid within the *Pospiviroidae* family, causes apple scar skin disease characterized by distinctive scar-like lesions on fruit surfaces, significantly reducing marketability (50, 51). This viroid has been reported in major apple-producing regions worldwide, with infection rates varying considerably between cultivars and geographic locations (52, 53). Beyond apple, ASSVd also naturally infects pear and quince, causing fruit deformities and quality reduction (54). In peach, *Prunus persica,* a particularly important *Rosaceae* crop, *Peach latent mosaic viroid* (PLMVd) represents the primary viroid pathogen (55). Unlike most known viroids, PLMVd belongs to the *Avsunviroidae* family and replicates in chloroplasts through a symmetric rolling-circle mechanism facilitated by hammerhead ribozymes (56). PLMVd infections typically remain latent for several years before symptom development, which includes mosaic patterns on leaves, fruit deformities, and delayed ripening (55, 57). Particularly, PLMVd variants induce an extreme chlorosis known as “peach calico,” characterized by severe albinism in affected leaf areas due to ribosomal stress and chloroplast development disruption (58). Molecular analysis has revealed that a specific structural element within these severe PLMVd variants, termed the “pathogenicity determinant,” is responsible for this dramatic symptomatology (59). *Apple dimple fruit viroid* (ADFVd), first identified in Italian apple orchards, causes fruit dimpling and deformation in susceptible cultivars (60, 61). Molecular characterization placed ADFVd within the *Apscaviroid* genus, showing moderate sequence homology with ASSVd despite distinct symptomatology (53).

The various transcriptomic and metatranscriptomic approaches have been used to identify viroids in various plant species (16, 19, 47). These include high-throughput next-generation library preparation followed by bioinformatics analysis. So far, various virus detection pipelines have been designed to detect covalently closed circular RNAs (62), such as viroids and viroid-like agents, including ambiviruses (63) and hepatitis delta virus relatives (64). Additionally, advanced bioinformatic algorithms have revolutionized viroid research, accelerated the discovery of novel viroid species and variants, revealing an unexpected diversity within these minimal pathogens. In this study, we analyzed ribosomal RNA-depleted transcriptomic data from grapevine, solanum, and rose plant families to detect previously uncharacterized viroids and viroid-like RNAs. We discovered two new viroid-like RNAs: the *Grapevine peach latent mosaic hammerhead viroid-like RNA* (280 nt), which has a branched architecture and a distinct hammerhead ribozyme, and the rod-structured *Cape gooseberry hammerhead viroid-like* RNA (274 nt), which shares conserved motifs with *avocado sunblotch viroid*. In peaches, we identified a new twister ribozyme-containing ex-circRNA exhibiting high transcriptional abundance and strand-specific self-cleavage activity. Additionally, three ambi-like circular RNAs encoding divergent RNA-dependent RNA polymerases, hairpin, and delta virus ribozymes were found in grapevine and peach. Furthermore, a novel Obelisk-like RNA with unique structural features was also detected in Cape gooseberry. These results highlight the overlooked complexity and evolutionary diversity of plant-associated subviral RNAs.

## RESULTS

### Characterization of ex-circRNAs across three host families

Starting with 57 RNA-seq libraries (185 Gb), 60 RNA-seq libraries (205 Gb), and 104 RNA-seq libraries (584 Gb) from the *Rosaceae*, *Vitaceae,* and *Solanaceae* SRA data sets, respectively, we performed transcriptome assembly followed by circular RNA detection using *vdsearch*, a tool designed for reference-free *de novo* synthesis of viroids and viroid-like sequences (62). This process resulted in the detection of 5,169, 36,557, and 29,770 initial circular RNA contigs, respectively (Table S1), yielding a combined total of 64,758 assembled contigs. The length distribution of these contigs offers important insights into the potential identity of viroid-like circular RNAs, thus out of the assembled total 64,758 contigs from all three host families, 63,358 were retained after size filtration (100–5,000 nt) ex-circRNAs. We reduced redundancy among the sequences by filtering and clustering them at 90% nucleotide identity using the MMseqs2 tool. Only clusters meeting the 90% identity threshold were retained for further analysis. Ultimately, we obtained 31,632 sequence clusters.

**Fig 1:**
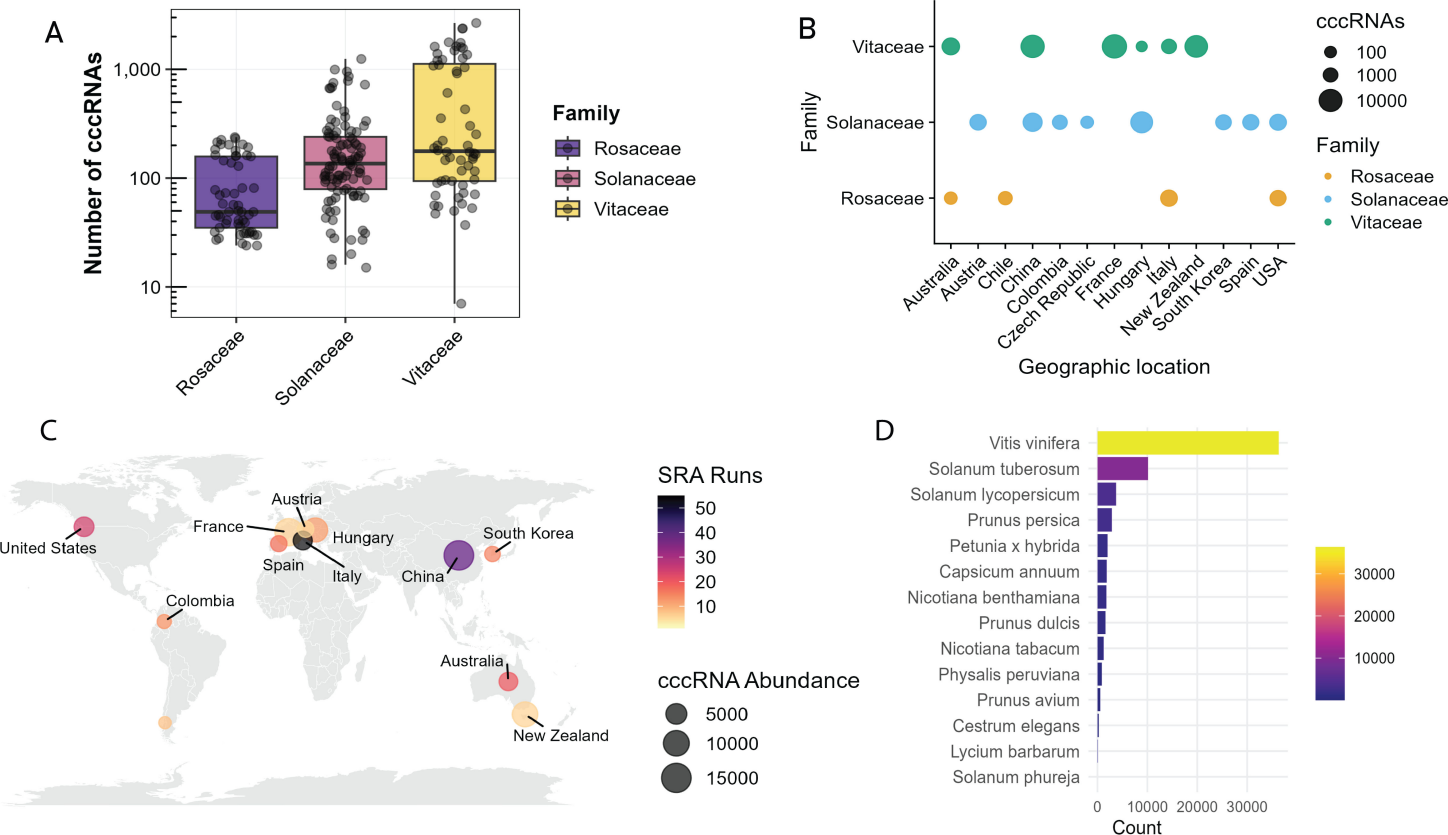
Summary of differential abundance of both exogenous and endogenous circular RNAs (circRNAs) across three plant families (*Vitaceae, Solanaceae,* and *Rosaceae*). (**A**) Log-scale distribution data of three families with *Vitaceae* displaying both higher median values and greater variance compared with *Solanaceae and Rosaceae* (*P* < 0.05). (**B and C**) The geographic distribution indicates sampling bias toward specific regions, with circle diameter proportional to circRNA abundance. (**D**) The total circRNAs detected in species of all three families. *V. vinifera* as the dominant species for circRNA content, followed by *S. tuberosum*, while remaining taxa demonstrate significantly lower abundances (*P* < 0.001).

The total number of ex-circRNA contigs detected in each SRA run from all these three host families (Fig. 1A; Table S1) by *vdsearch* showed that *Vitaceae* exhibits the highest median abundance (approximately 150 ex-circRNAs) among SRA runs, followed by *Solanaceae* (approximately 100 ex-circRNAs), and *Rosaceae* with the lowest median (approximately 50 ex-circRNAs) per SRA run. Notably, *Vitaceae* also shows the widest distribution range, with some samples exceeding 1,000 ex-circRNAs per SRA run, while others contain fewer than 10 (Table S1). To better contextualize these findings, we examined the associated metadata for each RNA-seq library. The *Vitaceae* data sets (*n* = 60), primarily collected from Australia, China, France, and New Zealand, consistently showed higher ex-circRNA counts compared with *Solanaceae*, despite consisting of fewer SRA runs (Fig. 1B and C). In contrast, the *Solanaceae* libraries (*n* = 104) were sourced from a broader range of countries, whereas the *Rosaceae* libraries (*n* = 57) originated mostly from Australia, Chile, Italy, and the USA. At the species level, *Vitis vinifera* (grapevine) from the *Vitaceae* family demonstrated the highest ex-circRNA abundance, contributing approximately 30,000 contigs, significantly surpassing *Solanum tuberosum* (potato) and other *Solanaceae* species (Fig. 1D). Although most of the analyzed species belong to the *Solanaceae* family, they generally exhibited lower levels of ex-circRNA accumulation relative to *Vitis vinifera*.

Using both homology-based and homology-independent methodologies (19, 62, 65, 66), we identified 16 previously known viroids and uncovered 13 novel circular RNAs (ex-circRNAs) that exhibit key features such as ribozyme activity, conserved structural motifs, and potential coding sequences (Tables S2 and S3). Read abundance analysis supports the reliable presence of these ex-circRNAs across samples (Fig. S1; Table S4). Of the 13 novel ex-circRNAs, six exhibit only partial features commonly associated with known viroids or viroid-like RNAs, such as lacking essential structural or sequence motifs (Fig. S2), and were therefore classified as unclassified ex-circRNAs at this stage. In the following sections, we provide a detailed characterization of the remaining ex-circRNAs that share multiple defining features with known viroids, organized by category.

### Detection of known viroids and viroid-like RNAs based on sequence homology

**Fig 2:**
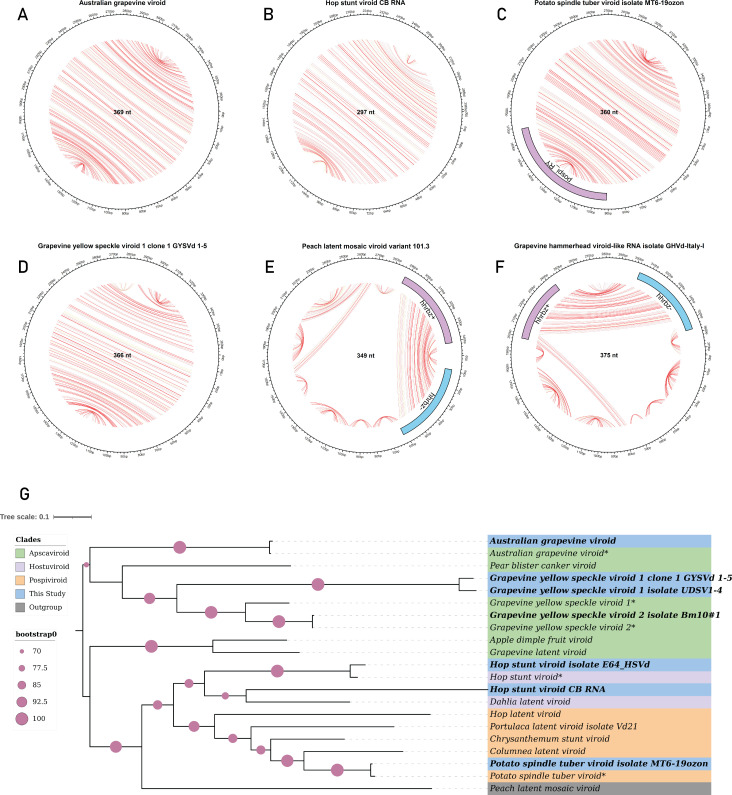
Circular RNA organization representation of six known viroid and viroid-like RNAs, highlighting predicted intramolecular base pairing interactions and phylogenetic analysis. (**A–F**) Circular genomes represented for *Australian grapevine viroid* (369 nt), *Hop stunt viroid* CB RNA (297 nt), *Potato spindle tuber viroid* isolate MT6 19ozon (360 nt) (Pospi_RY motif shown in purple), *Grapevine yellow speckle viroid 1, Peach latent mosaic viroid* variant 101.3 (349 nt), and *Grapevine hammerhead viroid-like* RNA isolate GHVd-Italy-I (375 nt) containing symmetric hammerhead motifs. The predicted base pairs are indicated by chords (base pair probabilities range from yellow, 0.1, to red, 1.0). (**G**) Phylogenetic relationships of known detected Pospiviroids (highlighted in bold), and current RefSeq viroid sequences available in GenBank for the family *Pospiviroidae*. The phylogenetic tree was built with 1000 bootstrap replicates using the maximum likelihood method. A color code was used to represent the different genera within this family. *Peach latent mosaic viroid* (PLMVd) (family *Avsunviroidae*) was included as an outgroup. Circles on branches indicate bootstrap values > 70% (generated from 1,000 replicates). Stars (*) represent reference pospiviroids.

To identify known viroids and viroid-like RNAs, we conducted a homology-based search of sequence clusters against a curated reference viroid database. Contigs with greater than 80% alignment coverage were further validated through BLASTn searches against the NCBI nucleotide (nt) database, leading to the recovery of multiple full-length or near full-length viroid sequences with high sequence identity (Fig. 2A through F; Fig. S3; Table S2). Within the *Vitaceae* family, we detected two variants of *Hop stunt viroid* (HSVd). The first variant, HSVd CB RNA (GenBank: LC500205.1), exhibited 99.6% identity over a 256-nt alignment with 86.2% coverage and an E-value of 2.71e-134, indicating near-complete homology with strong statistical support. The second variant, HSVd isolate E64_HSVd (GenBank: KJ466332.1), showed 100% identity over a 300-nt alignment, although with lower coverage (49.8%; E-value: 3.18e-154), suggesting a high degree of sequence similarity despite partial alignment. In addition, we identified *Australian grapevine viroid* (GenBank: MH476217.1) with 98.7% identity across 331 nucleotides (89.4% coverage; E-value: 9.84e-171), confirming robust sequence conservation in grapevine hosts. We also recovered two variants of *Grapevine yellow speckle viroid 1*. The first, GYSVd-1 clone 1 (GenBank: DQ371469.1), exhibited 100% identity over 303 nucleotides with 82.8% coverage (E-value: 9.25e-161), whereas the second, GYSVd-1 (GenBank: MF576400.1), also showed 100% identity over a 366-nucleotide alignment (E-value: 9.76e-190), reflecting strong conservation in grapevine samples. Furthermore, *Grapevine yellow speckle viroid 2* (GenBank: JQ686716.1) was recovered with 100% identity and 96.7% coverage (E-value: 2.84e-192), suggesting the presence of highly conserved isolates across *Vitaceae* data sets.

From 104 *Solanaceae* SRA data sets, we identified contigs corresponding to *Potato spindle tuber viroid* (GenBank: KY522733.1), which aligned with 98.7% identity across 325 nucleotides (90.3% coverage; E-value: 6.08e–167), indicating nearly full-length detection consistent with replication in potato hosts. Among the 57 *Rosaceae* data sets, we exclusively detected variants of *Peach latent mosaic viroid* (PLMVd). PLMVd isolate v1.10 (GenBank: KX430161.1) showed 95.8% identity across 334 nucleotides (E-value: 6.86e–152), whereas PLMVd variant 101.3 (GenBank: DQ680763.1) exhibited 97% identity over 335 nucleotides with 95.7% coverage. Additional variants included PLMVd clone PL01 (GenBank: HM185107.1) with 90.7% identity across 216 nucleotides (84.4% coverage; E-value: 6.82e–82), PLMVd isolate g7-p1 (GenBank: MK212088.1) with 95.8% identity over 337 nucleotides (E-value: 1.81e–153), and PLMVd clone P1.148-C349-g71 (GenBank: JN377866.1) with 96.7% identity and 338-nucleotide alignment, showing 49.1% query and 96.8% reference coverage (E-value: 1.21e–158), all indicating high sequence fidelity.

In addition, we identified several shorter contigs with partial alignment to PLMVd, suggesting incomplete or degraded viroid variants. These included PLMVd isolate V209 (GenBank: JF927895.1) with 80.6% identity over a 145-nucleotide alignment (E-value: 3.69e–33), covering 75.5% of the query and 43.0% of the reference; PLMVd isolate T01-11 (GenBank: MH974828.1) with 90.3% identity over 93 nucleotides (51.1% coverage; E-value: 9.46e–31); and PLMVd isolate ZZ36 (GenBank: JF898822.1) with 94.2% identity over a truncated 70-nucleotide alignment (26.8% coverage; E-value: 9.66e–25). We also recovered an unclassified viroid-like RNA, *Grapevine hammerhead viroid-like RNA isolate* GHVd-Italy-I (GenBank: KR736334.1), with 98.8% identity over 349 nucleotides (93.1% coverage; E-value: 3.41e–183), consistent with near-complete genome organization (Fig. 2F; Table S2).

To better understand the evolutionary relationships of the recovered viroid sequences, we constructed phylogenetic trees based on sequences from the *Pospiviroidae* and *Avsunviroidae* (Fig. 2G and 5A) family. The tree, generated using the maximum likelihood method with 1,000 bootstrap replicates, showed that the high-confidence viroid sequences recovered in this study clustered closely with their respective reference sequences, supporting their taxonomic classification and evolutionary relatedness.

### Discovery of a novel viroid-like RNA in *Physalis peruviana* with a symmetric hammerhead ribozyme

**Fig 3:**
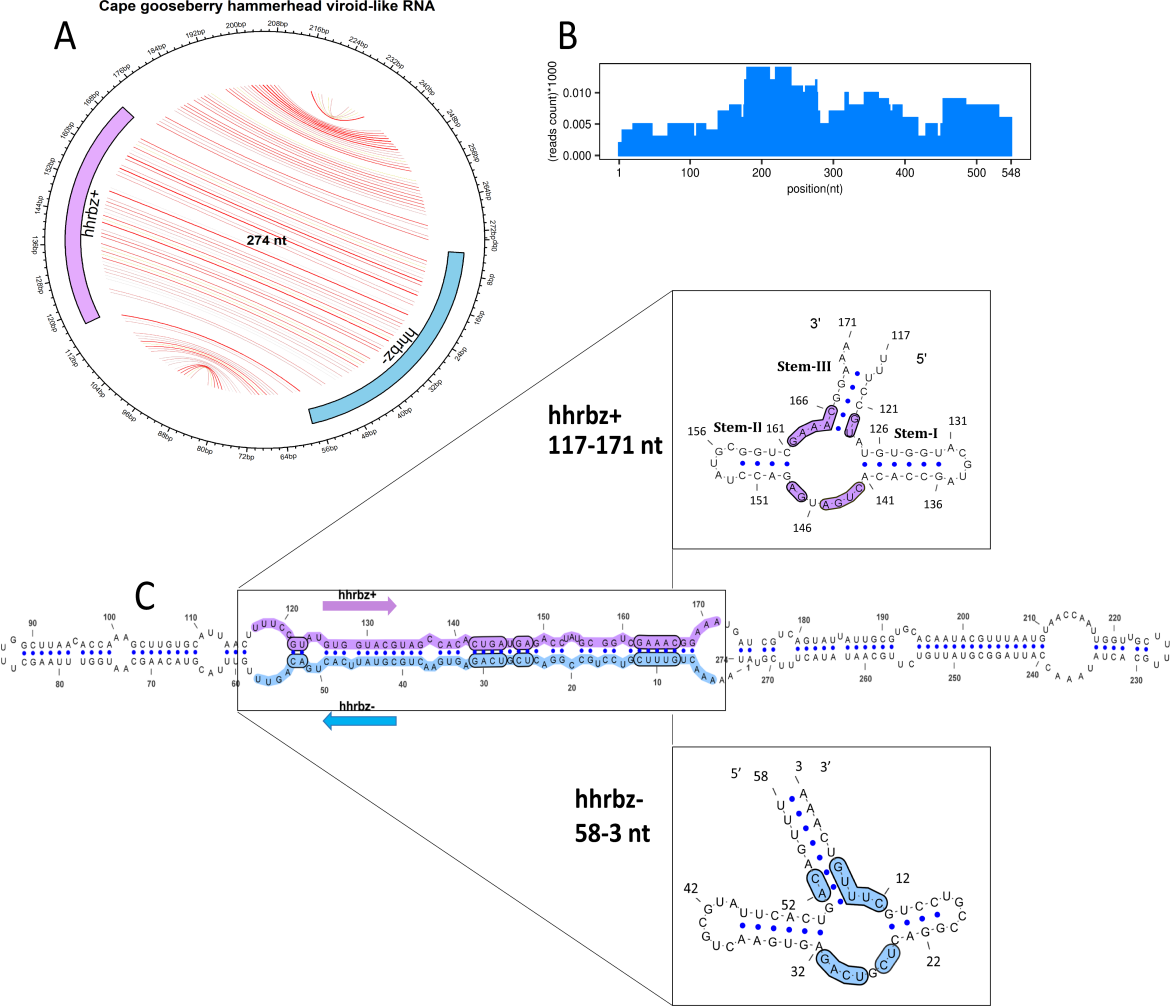
Characterization of *Cape gooseberry hammerhead* viroid-like RNA. (**A**) Circular plot of novel *Cape gooseberry hammerhead viroid-like* RNA (CGHVd-RNA) with 274 nt length. The predicted base pairs are indicated by bus chords (base pair probabilities range from yellow, 0.1 to red, 1.0) for all circular structures presented in this study. The symmetric hammerhead is shown at both the sense and antisense strands of the viroid. Purple color represents the plus strand (+) hhrbz, whereas blue color represents the negative strand (−) hhrbz. (**B**) histogram showing the reads mapped to the duplicated sequence of CGHVd-RNA, and (**C**) the RNAfold predicted minimum free energy quasi rod-like structure is shown with nucleotides and hhrbz highlighted in purple and blue as described earlier. Conserved domains of hhrbz+ and hhrbz− are shown magnified and highlighted.

In addition to a known viroid in the *Solanaceae* family, we also identified a new quasi rod-like viroid named *Cape gooseberry hammerhead viroid-like RNA* (CGHVd-RNA), consisting of 274 nucleotides with a G + C content of 41%. This viroid was detected in the Cape gooseberry plant (*Physalis peruviana,* Solanaceae) based on a significant alignment of this singleton cluster with the hammerhead III ribozyme (RF00008) from Rfam database, using the covariance model implemented in Infernal software, which also showed significant read coverage and depth (Fig. 3A and B). CGHVd-RNA did not exhibit sequence similarity or homology to any known viroids when searched against the NCBI nucleotide (nt) database using BLAST. The symmetric ribozymes were predicted, showing a rod-like secondary structure of symmetric hammerhead III ribozyme with stems I, II, and III and the conserved domains, which was also confirmed by the alignment-free method of ribozyme search using the RNABOB program (Fig. 3C). The first hammerhead ribozyme (hhrbz), denoted as hhrbz+ (56 nt), is located approximately between nucleotide positions 117 nt and 171 nt. This region exhibits the classical three-stemmed architecture characteristic of hammerhead ribozymes. Stem I initiates around nucleotide 120, extending into a short-paired region, whereas Stem II, forming the central conserved catalytic core, lies between roughly 135 nt and 145 nt. Stem III completes the motif, extending toward position 171 nt. The hhrbz− spans approximately positions 3–58 nt in reverse orientation. This minus-strand hammerhead ribozyme also displays a mirrored version of the plus-strand canonical structure with three distinct stems, aligning with the conserved hammerhead fold. A clearly discernible loop near the 5′ end (around nucleotides 1–10) is observed, representing part of Stem I or III, which forms an essential component of the folding topology required for self-cleavage activity. Together, the presence of these two oppositely oriented ribozymes suggests a symmetrical ribozyme arrangement typical of autocatalytically processed circular RNAs, supporting their classification as viroid-like replicons.

### Characterization of a highly divergent hammerhead viroid-like RNA in grapevine

**Fig 4:**
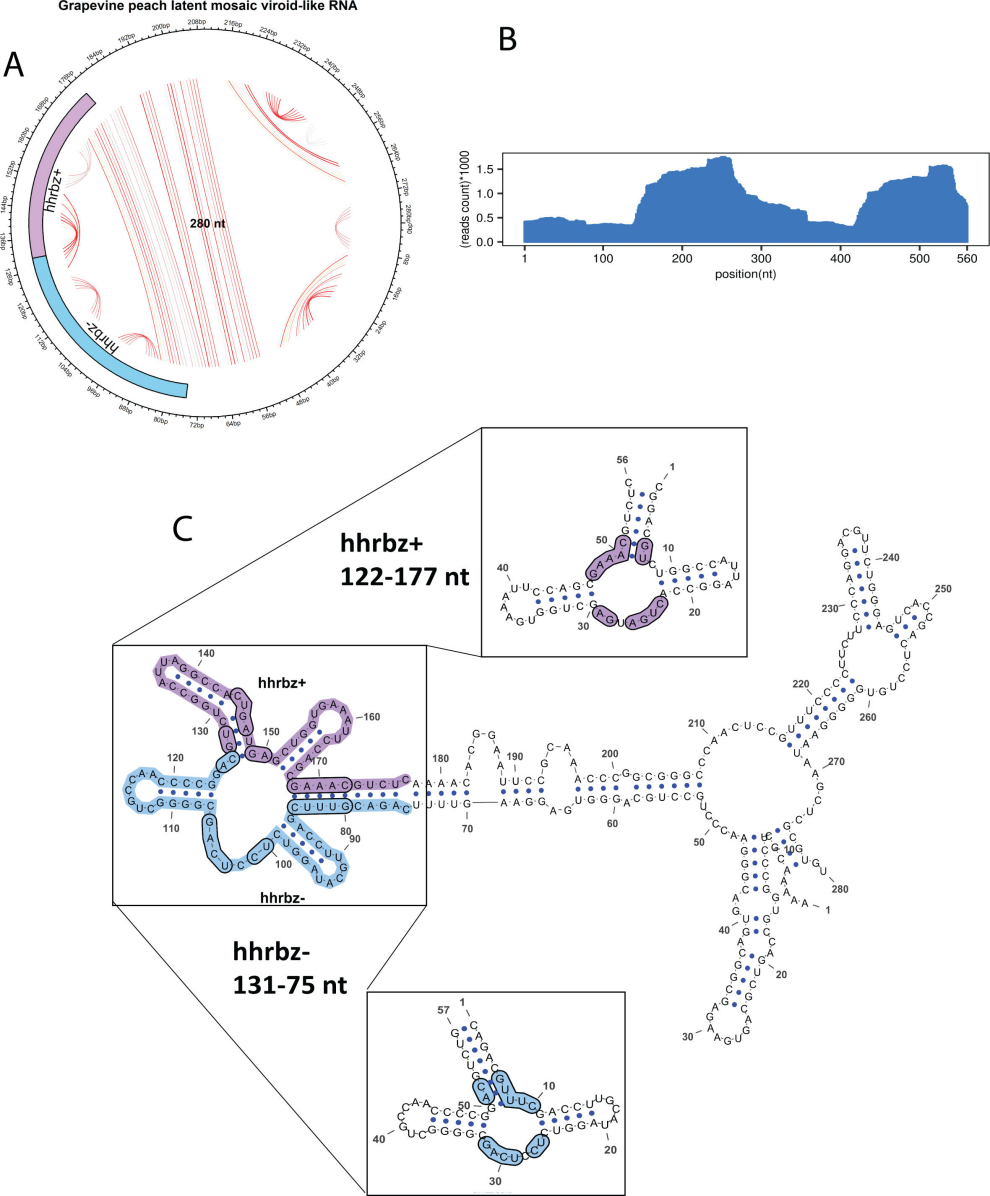
The structural organization of *Grapevine peach latent mosaic hammerhead* viroid-like RNA and read abundance. (**A**) Circular plot of Grapevine *peach latent mosaic hammerhead* viroid-like RNA (PLMHVd-RNA) with 280 nt length. The symmetric hammerhead is shown at both the sense and antisense strands of the viroid. Purple color represents hhrbz+, whereas blue color represents hhrbz−. (**B**) Histogram showing the reads mapped to the duplicated sequence of PLMHVd-RNA. (**C**) The RNA-fold predicted minimum free energy quasi rod-like structure is shown with nucleotides and hhrbz highlighted in purple and blue as described earlier. Conserved domains of hhrbz+ and hhrbz− are shown magnified and highlighted.

Previously, more common Pospiviroids have been reported in this plant family, that is, Grapevine latent viroid, *Grapevine yellow speckle viroids* 1 and 2 (the latter two are also detected in this study), etc. Additionally, one hammerhead containing viroid-like RNA, named *Grapevine hammerhead viroid-like* RNA, has also been reported but is yet to be recognized as a separate species of *Avsunviroidae* (19). The Grapevine PLMV-like RNA (GPLMVd-RNA) is a 280-nucleotide RNA with a G + C content of 58.93% (Fig. 4A), reported here for the first time in the *Vitaceae* (grapevine) family. Based on direct sequence homology search using the MMseqs tool against the reference viroid database, GPLMVd-RNA showed weak alignment to *Peach latent mosaic viroid* isolate PC-P1.148.94 (GenBank: DQ222096.1), which has a genome length of 336 nt. The alignment spanned only 47 nt (likely at the ribozyme region), with 16.8% coverage and an E-value of 1.25E-04, mapping query positions 349–173 nt to reference positions 7–51, suggesting marginal sequence similarity. Additionally, mapping RNA-seq reads to GPLMVd-RNA revealed high sequencing coverage, further confirming the presence of GPLMVd-RNA in the grapevine host (Fig. 4B).

GPLMVd-RNA shows a branched secondary structure with a symmetric hammerhead ribozyme III, as aligned to the hammerhead III ribozyme at the plus strand by ribozyme search against the Rfam database, using the Infernal program’s cmsearch module, thus showing characteristic structural homology to Genus *Pelamoviroid* (Fig. 4C). The presence of the same ribozyme was also confirmed using an alignment-free ribozyme search using RNAbob and RNAmotif search program independently. The depicted RNA structure represents a viroid-like circular RNA, more specifically characteristic of the *Peach latent mosaic viroid* (PLMVd) or a closely related PLMVd-like entity. The sequence folds into a branched secondary structure, a characteristic arrangement found in the *Avsunviroidae* family.

Several long stems formed by extended base-pairing, notably nucleotides 1–50 and 220–264, the multiple stem-loops and hairpins that stabilize the structure. These are crucial for the viroid’s structural integrity and interaction with host factors. The region from ~180–200 appears as a connecting single-stranded linker joining the ribozyme domain to the longer rod-like backbone. The hammerhead ribozyme on the positive-sense strand (hhrbz+: 122–177 nt), and its complementary counterpart on the negative-sense strand (hhrbz−: 131–75 nt) constitute a typical hammerhead ribozyme with three separate stem areas positioned just adjacent and opposite (Fig. 4C). Stem I starts around nucleotide 122 nt and proceeds in the direction of the central catalytic junction (GUCC) of hammerhead. Stem II extends from nucleotides 131–142 nt, and Stem III concludes the structure at approximately nucleotide 177 nt. A small loop is present in this ribozyme between positions 122 and 130, which is likely the intersection of Stems I and II. The loop provides the conformational flexibility necessary for folding into the catalytically active tertiary structure. Functionally, this ribozyme catalyzes self-cleavage of the positive-sense strand in the process of replication and hence enables the production of unit-length monomers from oligomeric intermediates of extended length.

On the other hand, the hhrbz− motif is oriented in a direction opposite to the strand, covering a region of about nucleotides 131–75 (Fig. 4C). This similar ribozyme structure also contains three helical stems surrounding a conserved catalytic core. The predicted structure exhibits the canonical three-stem hammerhead ribozyme architecture, characterized by three helices radiating from a conserved catalytic core. Stem I, forming the left arm of the motif, comprises base pairs extending approximately from nucleotides 131 to 114, stabilizing the region near the catalytic junction. Stem II, which appears as the vertical arm, includes base pairs spanning nucleotides 75–84, forming a continuous helix that extends toward the 3′ end. Stem III, positioned as the lower arm, encompasses base pairs between nucleotides 94 and 104. This hhrbz− facilitates the self-cleavage of the negative strand, thus ensuring that both polarities of the circular RNA can be efficiently processed during the entire symmetric rolling-circle replication process. Encircling these catalytic domains, the rest of the RNA adopts a tightly base-paired rod-like structure characterized by various stem-loops, internal bulges, and terminal hairpin formations. A notable multi-hairpin structure is seen around nucleotides 220–260, branching off from the central backbone and possibly playing a role in interaction with host cell factors or helping in the stability of the RNA.

### Phylogenetic analysis of CGHVd-RNA and GPLMVd-RNA reveals evolutionary divergence within the *Avsunviroidae* family

**Fig 5:**
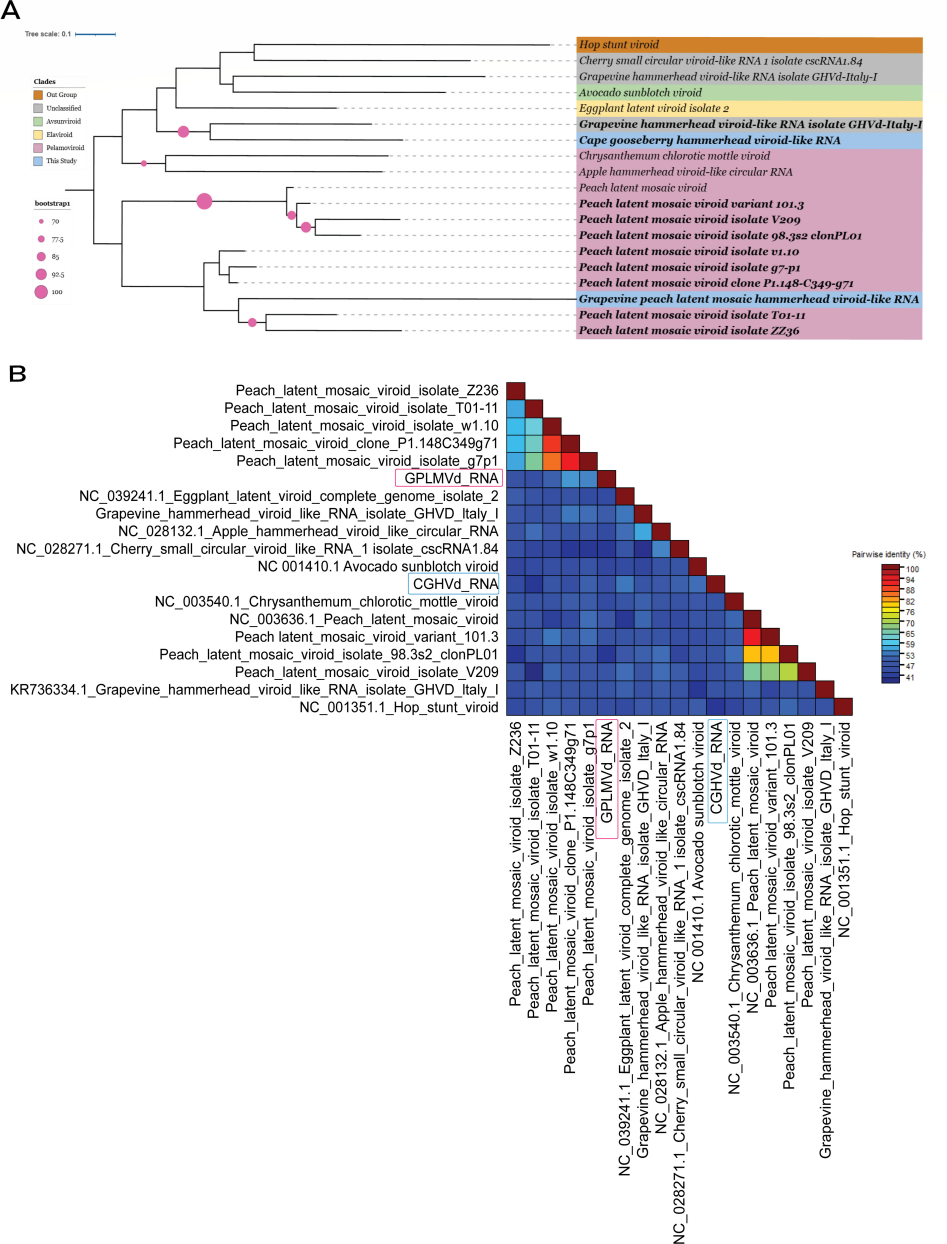
The phylogenetic tree and pairwise sequence alignment of newly discovered viroids in *Avsunviroidae*. (**A**) *Avsunviroidae* viroid genomic sequences were used to build maximum-likelihood phylogenetic trees. Red circles show bootstrap values >=70%, indicating that the tree is midpoint-rooted. This family’s several genera were represented by a color code. As an outgroup, the *Hop stunt viroid* (HSVd) (family Pospiviroidae) was included. The tree was visualized using the iTol web server (67). The bold labels are found in the current study. The newly discovered viroids in the study have a bluish hue. (**B**) Sequence Demarcation Tool (SDT) displays the PWISs (68). Sequence identities range from 0% (dark blue) to 100% (dark red) (corresponding from 0 to 1), as indicated in the color scale bar (right).

To elucidate the evolutionary relationships of CGHVd-RNA, GPLMVd-RNA, and *Peach latent mosaic viroid* (PLMVd) isolates from *Rosaceae*, we performed a comprehensive multiple sequence alignment incorporating all available RefSeq viroid sequences from the *Avsunviroidae* family in GenBank. A phylogenetic tree was then constructed using the maximum likelihood method implemented in IQ-TREE with 1,000 bootstrap replicates to assess branch support. Genus-level taxonomic distinctions within *Avsunviroidae* were highlighted using chromatic markers, and the analysis included unclassified hammerhead ribozyme-containing viroid-like RNAs to provide a broader evolutionary context (Fig. 5A). The resulting tree revealed that CGHVd-RNA shares only a distant relationship with known viroids, clustering weakly with an unclassified *Grapevine hammerhead viroid-like* RNA at a 70% bootstrap value. GPLMVd-RNA formed a highly divergent branch, showing no close relationship with existing members of *Avsunviroidae*. Notably, both CGHVd-RNA and GPLMVd-RNA represent novel host associations, as these viroid-like RNAs have not been previously reported in Cape gooseberry or grapevine.

The pairwise comparison-based results of SDT analysis (Fig. 5B) showed that the minimum and maximum pairwise identity (PWISs) demonstrated sequences of newly characterized viroids CGHVd-RNA and GPLMVd-RNA exhibited significant sequence divergence, showing less than 73% genus and 90% species level PWISs (69), respectively, with any previously documented viroid sequences across their complete genomes. Despite this genetic distinctiveness, CGHVd-RNA maintains the characteristic rod-like secondary structure with an embedded hammerhead ribozyme domain, a structural motif consistent with members of the *Avsunviroidae* family. This combination of novel sequence composition with conserved structural elements suggests these isolates represent potentially new taxonomic entities within the viroid classification system.

### Identification of symmetric twister ribozyme-containing circular RNAs in peach

**Fig 6:**
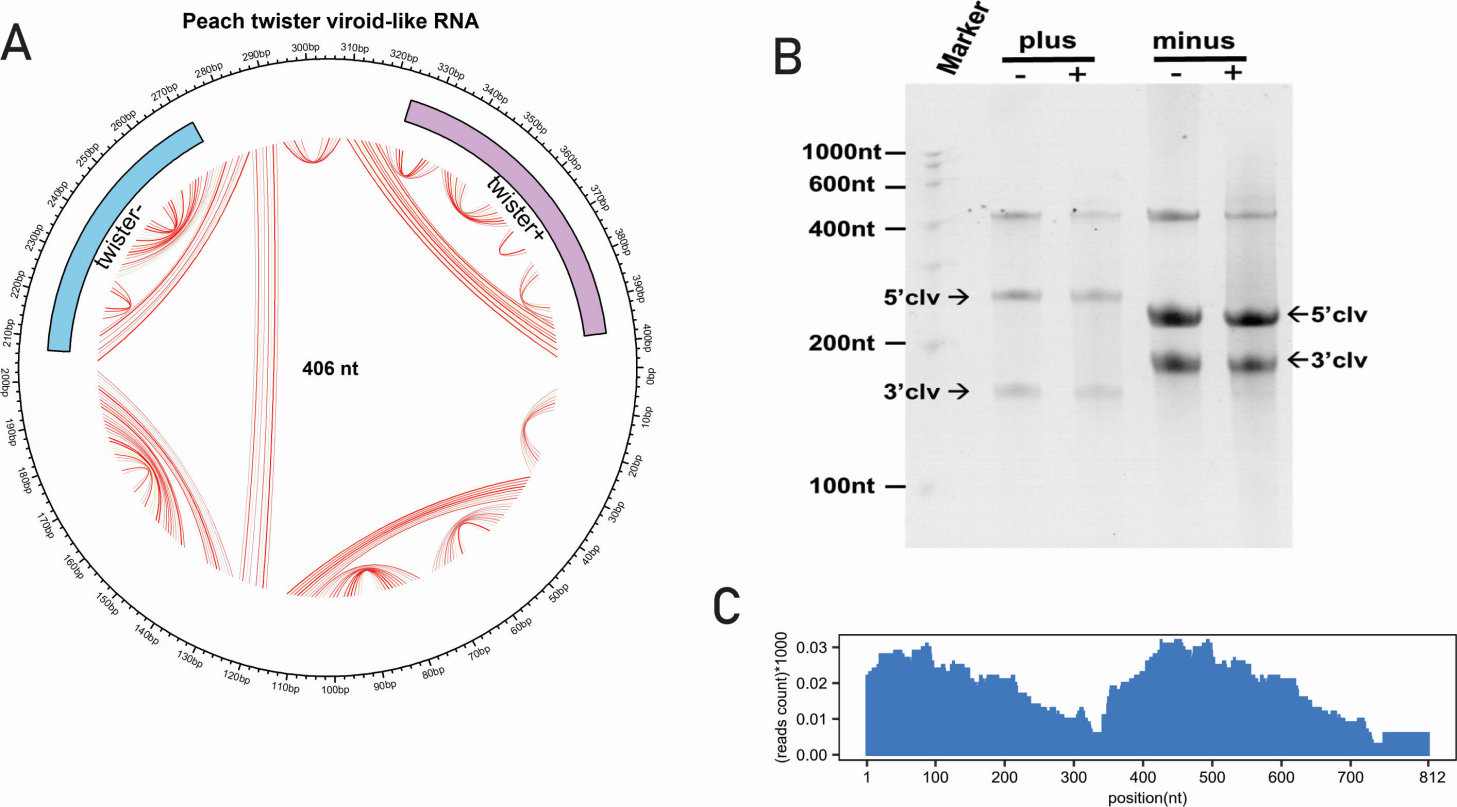
The characterization of novel twister ribozyme containing cccRNAs. (**A**) Circular plot of *Peach twister viroid-like* RNA with 406 nt length. The genomic organization is depicted in Fig. 3A. The symmetric twister-P1 is shown at both the sense and antisense strands. Purple color represents tw+, whereas blue color represents tw-. (**B**) *In vitro* transcription self-cleavage reactions of purified monomeric products were separated by 5% denaturing polyacrylamide gel electrophoresis and visualized by ethidium bromide staining, indicating plus (-/+) and minus (-/+) monomers of complete transcripts of *peach twister viroid-like RNA*. (**C**) The histogram of reads mapped to the duplicated genome shows abundance at ribozyme sites.

The presence of twister ribozyme in plants (70) has been reported previously and extensively studied in rice plants (71), but the symmetric twister containing ex-circRNAs with symmetric ribozyme presence has not been reported in the *Rosaceae* family. Here, we reported new twister containing ex-circRNA in peach (*Prunus persica*) plants (SRA No.: SRR17074236), we named it *Peach twister viroid-like RNA* (406 nt with G + C content 58.13%) (Fig. 6A). This sequence cluster was detected through Rfam database screening using the Infernal tool and showed alignment to the symmetric twister P1 ribozyme (RF03160) on both strands: 324–398 nt (75 bp, E-value: 2e–10) on the plus strand and 272–207 nt (66 bp, E-value: 8.3e–13) on the minus strand (Fig. 6A). *In vitro* self-cleavage assays were conducted using T7 promoter-containing templates, followed by transcription and cleavage in specific buffer conditions. Urea-PAGE analysis confirmed that both plus and minus strands exhibited self-cleavage activity, with the minus strand showing significantly higher cleavage efficiency based on gray value analysis (Fig. 6B). Furthermore, RNA-seq read mapping revealed notable read abundance in the peach host, supporting the biological relevance of this RNA species (Fig. 6C).

### Identification of ambi-like circular RNA viruses in grapevine and peach

**Fig 7:**
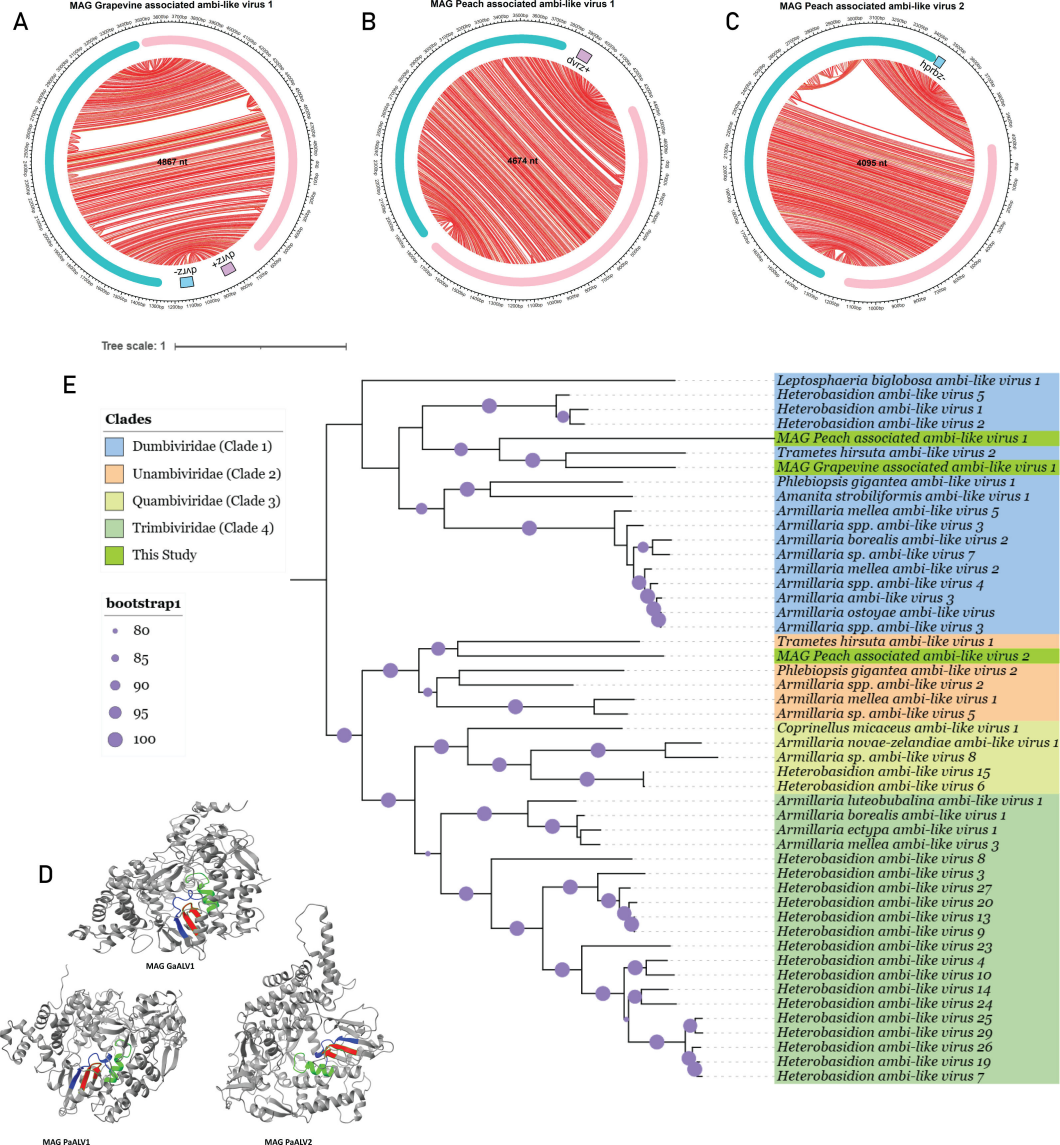
The characterization of ambi-like cccRNAs discovered in grapevine and peach SRA runs. (**A–C**) The circular organization of the ambi-like viruses. The red and green bars indicate the range of the two predicted ORFs, which are similar to the RdRP and hypothetical protein of known ambi-like viruses for MAG GaALV1, MAG PaALV1, respectively, but in the case of MAG PaALV2, the green represents RdRP. (**D**) Predicted RdRP structures by Alphafold2 showing palmprint motif A(blue), B(Green), and C(Red) for MAG GaALV1, MAG PaALV1, and MAG PaALV2, respectively (Table S6). From red (low confidence) to blue (very high confidence), colors represent a measure of confidence in the local structure (pLDDT). (**E**) Maximum likelihood Phylogenetic tree using ultrafast bootstraps (1,000), and LG  +  G4  + I best-fit model.

Ambiviruses have circular genome around ~4–5 kb with a rod-shaped ssRNA structure comprising hhrbz or Delta virus ribozymes (DVRz) with two ORFs recently discovered abundantly in fungi (63, 72) and are thought to be the largest circular RNA genomes known yet. In this study, three ambi-like viruses encoding RdRP were detected at significant E-values. Their full-length circular genomes demonstrate two ORFs in sense and antisense orientation along with predicted ribozyme motifs (Fig. 7A). MAG *Grapevine-associated ambi-like virus 1* (MAG GaALV1), identified from grapevine libraries, has a circular genome of 4,867 nt and contains symmetric Delta virus ribozymes (DVRz) along with two ORFs. One ORF encodes an ambivirus-like RdRP consisting of 705 amino acids, whereas the other encodes a putative hypothetical core protein. BLAST analysis of the RdRP revealed homology to *Trametes hirsuta ambi-like virus 2*, with 38.36% coverage and a highly significant E-value of 5e–139. Based on these findings, we considered it a potentially novel ambi-like virus. The second ambi-like virus was identified from peach (*Rosaceae* family) SRA data using a homology-independent ribozyme search via the RNABOB program. DVRz descriptors (Fig. S4) (73) were used to detect potential DVRz motifs. This virus, named *MAG Peach-associated ambi-like virus 1* (MAG PaALV1), has a genome length of 4,674 nucleotides and features a single DVRz along with two ORFs. The RdRP, consisting of 687 amino acids, indicates a divergent RdRP and showed 99% query coverage and 68.62% identity to the RDRP of *Ambivirus sp*. (GenBank: DBA53192.1). Additionally, we identified another ambi-like virus with a distinct genome size and organization compared with the previously described ambi-like viruses, which we named *MAG Peach-associated ambi-like virus 2* (MAG PaALV2). Detected in *Prunus persica* (*Rosaceae* family), MAG PaALV2 has a genome length of 4,095 nt and a G + C content of 48.47% (Fig. 7C). Unlike MAG PaALV1, MAG PaALV2 contains hairpin ribozymes instead of delta virus ribozymes and features an RdRP-coding ORF spanning 704 amino acids located on the positive strand (~1,300–3,414 nt). The RdRP shows 100% query coverage and 91.05% identity to the RdRP of *Ambivirus sp*. (GenBank: DBA53168.1).

PalmScan is a software used to detect the catalytic motifs (e.g., motifs A, B, and C) in viral RdRPs, generating “palmprints” that can be used to classify RNA viruses (74). To assess their conserved polymerase features, we used AlphaFold2 to predict the 3D structures of all three RdRPs. PalmScan analysis confirmed the presence of the hallmark catalytic motifs A, B, and C across all proteins (Fig. 7D; Table S6), supporting their functional classification as RNA-dependent RNA polymerases. To explore their evolutionary relationships, we reconstructed a phylogenetic tree using RdRP sequences from this study alongside those of previously characterized ambi-like viruses (Fig. 7E). The phylogenetic analysis revealed distinct evolutionary patterns among the newly identified viral species (Fig. S5). Despite being derived from the same host species, MAG PaALV1 and MAG PaALV2 displayed considerable genetic divergence, with MAG PaALV2 clustering within the *Unambiviridae* clade (Clade 2), whereas MAG PaALV1 clustered within the *Dumbiviridae* (Clade 1). This taxonomic distinction suggests independent evolutionary origins and potentially different biological properties or host interactions. Interestingly, GaALV1, identified in grapevine, was more closely related to MAG PaALV1 than to MAG PaALV2, indicating potential cross-host viral relationships or shared ancestral origins. Given the fungal origin of most known ambiviruses, we further analyzed the associated microbial communities in the metatranscriptomic libraries using Kraken2 (Fig. S6A through D; Table S7) and estimated the relative percentage of species in host families. The presence of diverse microbial taxa (Fig. S7A through C; Tables S8 to S10) suggests that these ambi-like viruses may originate from plant-associated holobionts rather than directly from the plant hosts themselves. However, definitive host associations remain unresolved, and further experimental validation is needed to determine their true biological hosts and assess their potential roles in host–virus interactions.

### Discovery of a structurally unique Obelisk-like circular RNA in Cape gooseberry

**Fig 8:**
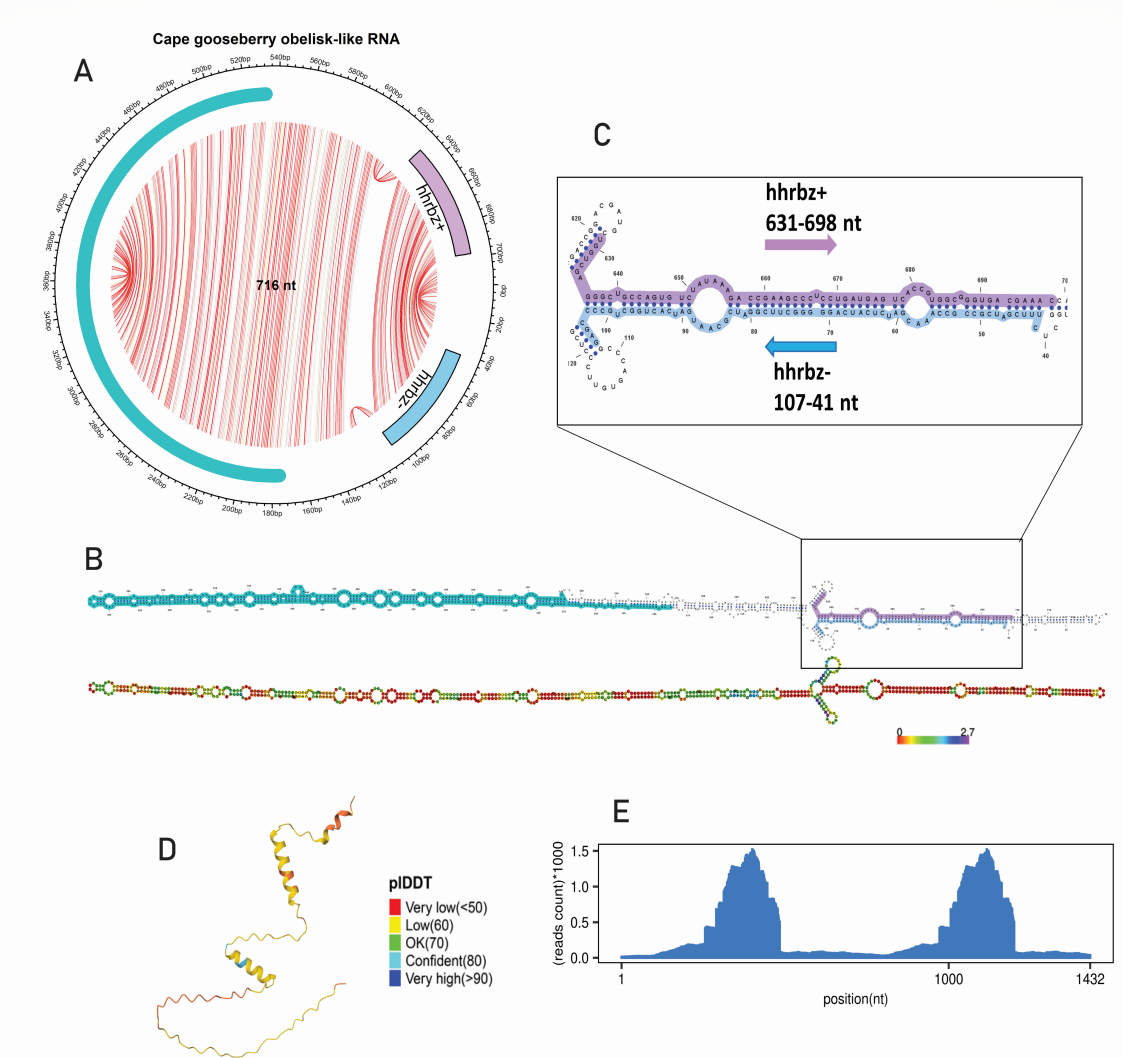
Genomic organization and characterization of *Cape gooseberry obelisk-like* RNA. (**A**) The circular predicted structure shown by the RNA-fold package with ribozyme and a red box for a single ORF. (**B**) Predicted rod-shaped secondary structure predicted by RNA-fold and a parallel Varna structure showing hhrbz (hammerhead ribozyme). (**C**) Within the extended boxed area, we can find the region where the ribozyme is located and its rod structure. (**D**) Predicted tertiary structure (AlphaFold2 using MMseqs2) (76, 77) of the ORF that lacks a good tertiary structure prediction. From red (low confidence) to blue (very high confidence), colors represent a measure of confidence in the local structure (pLDDT). (**E**) Read mapping abundance is shown in a red box for a duplicated sequence.

Obelisks were recently discovered in human gut microbiomes (65), followed by their abundant detection in oceanic microbiomes (75). These RNAs typically have a circular ∼1 kb RNA genome, rod-like secondary structures, and encode a protein superfamily known as “Oblins.” The Oblin-1 protein is predicted to have RNA-binding capabilities, as suggested by its tertiary structure and a domain enriched with positively charged residues, which may facilitate interactions with negatively charged molecules like RNA. Oblin-2 is hypothesized to function either as a homo-multimer or as a binding partner for host leucine zipper proteins (65). Some Obelisk variants also carry hammerhead ribozymes (hhrbz) of the Obelisk-variant type III (ObV-HHR3) on both polarities (65, 75). In this study, we identified an obelisk-like circular RNA (716 nt) in Cape gooseberry RNA-seq data, which we named Cape gooseberry obelisk-like RNA (Fig. 8A). This molecule was detected using a homology-independent, ribozyme-based search with RNAbob descriptors targeting hammerhead ribozymes, as it exhibited no sequence or structural homology to any known circular RNA in public databases.

The predicted secondary structure revealed a rod-like fold, with a hammerhead ribozyme on the positive strand spanning 631–698 nt (67 nt in length) and another on the negative strand spanning 107–140 nt near the sequence’s 5′ end (Fig. 8B and C). A single ORF (176–532 nt) was predicted, encoding a 118-amino-acid protein that showed no sequence homology to any known proteins in the NCBI non-redundant (nr) database (Fig. 8A). Structural prediction using AlphaFold2 revealed that the protein does not align with either Oblin-1 or Oblin-2 motifs found in previously described Obelisks (Fig. 8D). However, the RNA shares two hallmark features of Obelisks: a rod-shaped secondary structure and the presence of hammerhead ribozymes similar to ObV-HHR3. Moreover, RNA-seq read mapping showed notable read abundance in the host library, supporting the presence of this circular RNA in Cape gooseberry (Fig. 8E). These results suggested that this RNA might represent either a divergent member of the eukaryotic Obelisks (75) or a new lineage within eukaryotic circular RNAs.

To further investigate the potential origin of this element, we performed taxonomic profiling of the Cape gooseberry data set using Kraken2. The analysis revealed the presence of diverse microbial communities (Fig. S7C; Table S10), raising the possibility that the RNA could originate from plant-associated microorganisms rather than the host plant itself. To assess whether the RNA might be of prokaryotic origin, we searched for CRISPR targeting signatures by querying the Joint Genome Institute’s IMG/M CRISPR spacer database. No significant matches were found across bacterial or archaeal genomes (BLASTn E-value <1e-5; minimum 85% identity over 20 nt), suggesting that this RNA has not been subjected to CRISPR-mediated immune surveillance. Although the absence of CRISPR targeting is consistent with a eukaryotic origin, it does not definitively exclude alternative sources. Further investigation will be required to clarify its biological role and evolutionary context.

## DISCUSSION

This investigation presents a comprehensive transcriptomic analysis of viroids and viroid-like exogenous circular RNAs (ex-circRNAs) across three major plant families: *Vitaceae*, *Solanaceae*, and *Rosaceae*. Employing both homology-based and homology-independent methodologies (19, 62, 65, 66), we identified known viroids with high confidence and discovered several novel circular RNAs exhibiting ribozyme activity, structural motifs, and potential coding sequences. These results reaffirm the extensive distribution of *Pospiviroidae* and *Avsunviroidae* members across diverse plant species and validate the efficacy of integrating secondary structure prediction (78) with Rfam-based ribozyme detection for streamlined viroid identification and surveillance.

Utilizing the robust vdsearch algorithm (62), we assembled 64,758 circRNAs from 221 paired-end raw sequencing libraries obtained from the SRA. Of these, 63,358 contigs exhibited lengths ranging from approximately 100 to 5,000 nt, with most sequences falling within the 200–800 nt range, which corresponds to the typical size range of viroid-like sequences (62, 63, 78, 79). This size distribution indicates substantial representation of viroid-like or viral elements and corroborates recent studies demonstrating the abundance and diversity of viroid-like RNAs in plant systems. A homology-based search enabled the identification of known viroids and viroid-like RNAs across the *Vitaceae*, *Solanaceae*, and *Rosaceae* families, leading to the recovery of multiple full-length or near full-length sequences with high identity. In *Vitaceae*, we detected several variants of *Hop stunt viroid, Australian grapevine viroid*, and *Grapevine yellow speckle viroid* types 1 and 2 with up to 100% identity and strong coverage. In *Solanaceae*, *Potato spindle tuber viroid* was identified with nearly complete sequence recovery. In *Rosaceae*, multiple *Peach latent mosaic viroid* variants were detected. Additionally, we recovered an unclassified *Grapevine hammerhead viroid-like RNA* with near-complete sequence identity with the reference genome.

Importantly, we report two novel viroid-like RNAs—CGHVd-RNA in *Physalis peruviana* (*Solanaceae*) and GPLMVd-RNA in *Vitis vinifera* (*Vitaceae*)—both of which contain symmetric hammerhead ribozymes and exhibit structural features characteristic of the *Avsunviroidae* family. However, these RNAs display limited sequence similarity with any known viroid species and fall below established taxonomic pairwise identity thresholds (<90%), suggesting that they may represent distinct evolutionary lineages within *Avsunviroidae*. Phylogenetic analyses support this conclusion, with both viroid-like RNAs forming separate branches within *Avsunviroidae*, divergent from known genera. This observation aligns with recent metatranscriptome analyses revealing that viroid-like circRNAs occupy a vast, understudied sequence space where structural motifs persist despite rapid sequence divergence (80). These discoveries expand the known host range of hammerhead viroid-like RNAs and point to greater structural and evolutionary plasticity within this group than previously understood. The structural diversity observed in these RNAs, particularly their retained ribozyme functionality despite low sequence conservation, supports the hypothesis that structural motifs are more evolutionarily stable than primary sequences in these pathogens, a pattern now recognized across diverse ecosystems (81).

We report the first identification and functional validation of a circular RNA containing symmetric twister ribozymes in peach (*Prunus persica*), marking a novel discovery and significant expansion of known ribozyme diversity within the *Rosaceae* family. This RNA, tentatively named *Peach twister viroid-like RNA* (406 nt), harbors P-1 class twister ribozymes (RF03160) on both strands with strong statistical support, aligned with a recent study in which computational predictions have identified hundreds of twister-containing cccRNAs (6, 82), with experimental validation remaining rare (80), making this functional characterization particularly valuable. *In vitro* self-cleavage assays have confirmed strand-specific catalytic activity, with the minus strand exhibiting higher cleavage efficiency (6, 82). This autocatalytic activity suggests a functional replication mechanism and suggests potential host interactions. These findings expand the known distribution of twister ribozymes in plant transcriptomes (81, 83) and raise the possibility that such ribozyme-associated circular RNAs may be more widespread and functionally relevant than previously recognized.

Additionally, we identified three large ambivirus-like circular RNAs (~4.5–5 kb) in grapevine and peach RNA-seq libraries, that is, MAG *Grapevine-associated ambi-like virus* 1, MAG *Peach-associated ambi-like virus* 1, and MAG *Peach-associated ambi-like virus* 2. These molecules resemble fungal ambiviruses in genome structure and encode divergent RdRPs (84). All three harbor hallmark features of fungal ambiviruses, including symmetric Delta virus ribozymes (in two cases) and asymmetric hairpin ribozymes (in the third). RdRP sequence analyses revealed partial similarity to known fungal counterparts—for instance, *MAG Peach-associated ambi-like virus 1* shared 68.62% similarity with the RdRP of *Ambivirus sp*. (DBA53192.1), whereas *MAG Grapevine-associated ambi-like virus 1* encoded an RdRP related to *Trametes hirsuta ambivirus* (E-value: 5e–139). Furthermore, we discovered an obelisk-like circular RNA (716 nt) in *Physalis peruviana*, exhibiting characteristic features such as circular topology, a rod-like secondary structure, and paired hammerhead ribozymes. Although it lacks sequence or structural homology to known ex-circRNAs, including the Oblin-1 and Oblin-2 domains (65, 75), its features are consistent with the broader Obelisk architecture. This finding reinforces the value of structure-based detection strategies, as highlighted in recent viroid discovery frameworks (62, 65), and underscores the limitations of homology-dependent methods in detecting rapidly evolving RNAs (81).

Taken together, our findings reveal a remarkable and largely unexplored diversity of subviral agents and ribozyme-bearing circular RNAs in plants. This study highlights the power of integrative transcriptomic analyses and ribozyme-centered computational approaches to uncover hidden elements of the plant RNA virosphere. Future research should focus on elucidating the biological functions, replication mechanisms, and potential pathogenicity of these novel cccRNAs.

## MATERIALS AND METHODS

### Data acquisition and quality analysis

The primary step for the in-silico discovery was to download the ribosomal RNA (rRNA) depleted Sequence Read Archive (SRA) raw transcriptomic and metatranscriptomic data in fastq format using software ascp from the EBI server (ftp://ftp.sra.ebi.ac.uk). We included 60 grapevine SRA libraries from *Vitaceae*, 105 SRA runs from *Solanaceae,* and 57 from *Rosaceae* plant families in which the epidemiology of viroids has previously been reported worldwide (Table S1). The paired-end raw reads were analyzed by Fastp (85) using default parameters and filtered to get clean reads. The Fastp detects the quality of reads, automatically detects adapters, and clips them to produce clean read files.

### *de novo* assembly of exogenous circular RNAs

Reference-free *de novo* assembly of paired FASTA reads was performed by *rnaspades* (86), and contigs were searched for the detection of viroids and viroid-like circular RNAs by a robust novel algorithm *vdsearch* (62) implemented in the NIM programming language, built specifically to seek covalently closed circular RNAs (i.e., ex-ccRNAs). After that, the clustering step was performed to reduce the redundancy of assembled circRNA genomes. For this purpose, assembled circRNAs were dereplicated at 90% nucleotide identity using mmseqs2 easy-linclust as described previously by many studies (87, 88). The representative sequence of each cluster was used for downstream analysis.

### Detection of viroids and viroid-like ex-circRNAs

Assembled ex-circRNAs were subjected to homology search against ViroidDB (89) and a hairpin ribozyme (HP) data set (90), implementing mmseqs2 easy-search (v. 13.45111) (87) with the maximal sensitivity parameters (-s 7.5). For each query sequence, only the highest-scoring alignment based on bit score was retained as the definitive match (62). Additionally, the assembled ex-circRNAs were screened for the presence of characterized self-cleaving ribozymes employing the covariance model search algorithm cmsearch (91) implemented in the Infernal software suite against the comprehensive Rfam structural RNA database (92, 93) with a significance threshold of E-value <0.1. Sequences exhibiting ribozyme motifs that satisfied these stringent criteria were classified as viroid-like exogenous circular RNAs.

Utilizing alignment-independent methodologies, we conducted targeted screening for sequences harboring statistically significant (E-value <0.01) hammerhead ribozyme structural motifs using specific descriptors for each ribozyme (Fig. S4). This analysis was executed via the RNAmotif algorithm (94), implementing descriptor files previously established and validated recently (62). We considered these sequences viroid-like because they contain hammerhead ribozymes, which are not reported before; hence, we assumed that these ribozymes are more divergent than the available ribozyme database. Additionally, we also used another software RNABOB (95) for the reference-free detection of hammerhead and HDV ribozymes. Additionally, we also separated sequences shown *Hepatitis delta virus* ribozyme (DVRz or dvrbz), as we know DVRz is a special class of ribozyme, which was previously described in metazoan species only; however, it has recently been predicted in ambiviruses of fungi (63) and human microbiomes (65). For this purpose, we used different descriptors for reference-free detection of such ribozymes (73, 96).

### Public homology searches using BLAST

To identify potential viroids and viroid-like ex-ccRNAs, the assembled circRNAs were searched for similar sequences in individual or grouped genomes that existed in the National Center for Biotechnology Information (NCBI) by Blast nt and nr (97, 98) databases using default parameters with the cutoff e-value 1e-03. For additional protein homology searches, we used translated BLASTX-style search against the UniRef90 protein database (99) using Diamond BlastX (100). For each ex-circRNA, we consider only the best match by e-value and score. A second approach, we applied hmmscan to search for the ORFs predicted by orfipy (101) from all ex-ccRNAs, against the Pfam-A database (102) using HMMER (103) program. To identify conserved RNA-dependent RNA polymerase (RdRp) palmprint motifs (A, B, and C) characteristic of potential ambiviral genomes, predicted ORFs were screened using Palmscan (74). Input amino acid sequences were supplied in FASTA format, and Palmscan was executed with default parameters to detect canonical palmprint motifs within the catalytic domain. Identified palmprint regions were manually inspected to confirm the presence and order of motifs A, B, and C, ensuring alignment with reference ambiviral RdRp signatures.

Due to their demonstrated sequence novelty, the Obelisk-α/β Oblin-1 and -2 protein sequences (65, 75) were subsequently employed as diagnostic molecular signatures specific to Obelisk-like RNAs—a systematic approach analogous to the utilization of RNA-dependent RNA polymerase (RdRP) sequences as taxonomic markers in RNA viral discovery pipelines (63, 74, 104) For comprehensive tertiary structural analysis of all open reading frames, AlphaFold3 was implemented with standard parameters (76), utilizing mmseqs2 *uniref env*. database for multiple sequence alignment generation, for quantitative assessment of Obelisk-like RNAs. Structural homology evaluation was conducted via FoldSeek (105) employing default algorithmic parameters. Visualization and analysis of the predicted protein tertiary structures were executed through the ChimeraX computational platform (106).

### Secondary structure prediction

For secondary RNA structures, we folded viroid and viroid-like ex-circRNAs into the minimum free energy structures with the RNAfold program in the Vienna RNA package with parameters, i.e -p, -r, -d2, --noLP, --circ, and setting temperature at 37°C (107). We used the p-num parameter to get circular plots of viroids and viroid-like ex-circRNAs. The right positions of ribozymes and any ORFs in the case of other viroid-like circRNA sequences were depicted by the *circlize* (108) package, an implementation for circular layout generation in R, and the Varna program (109) for secondary structure visualization.

### Analysis of plant holobiont using Kraken2/Bracken

Quality-filtered RNA-seq reads were directly subjected to taxonomic classification using Kraken2 (v2.1.2) (110) with a custom database incorporating bacterial, archaeal, fungal, viral, and plant reference genomes to characterize the complete holobiont community. The database was constructed using kraken2-build, incorporating RefSeq complete genomes for microbial taxa and plant genomes from NCBI RefSeq (111). Plants, with classification performed using default parameters and a confidence threshold of 0.1. Kraken2 taxonomic assignments were subsequently processed with Bracken (v2.6.0) (112) to estimate species-level relative abundances, applying a read length of 75 bp for Bayesian re-estimation, species-level taxonomic resolution, and a minimum threshold of 10 reads per taxon. Bracken analysis was conducted separately for each taxonomic domain to optimize re-estimation accuracy, redistributing reads initially assigned to higher taxonomic levels and generating refined abundance profiles for each holobiont component. Final taxonomic profiles species-level read abundance composition was consolidated to produce comprehensive holobiont compositions displaying relative proportions of plant host and associated microorganisms, as shown in Fig. S6A through C and Fig. S7A through C, visualized using R scripts.

### Phylogenomic analysis of detected viroids and viroid-like circRNAs

For identified new and known viroids, multiple sequence alignment (MSA) was performed using the MAFFT v7.310 program (113) independently with all RefSeq sequences from *Pospiviroidae* and *Avsunviroidae*, downloaded from the NCBI GenBank. Post-alignment, gaps were meticulously trimmed using the Trimal software (114) to reduce noise in subsequent analyses. The alignments were visualized by Jalview (115). The phylogenetic tree construction was executed using IQ-TREE (116) software for maximum likelihood analysis with options “-bb 1000 and -alrt 1000” to conduct 1,000 ultrafast bootstrap approximations and approximate likelihood-ratio test (aLRT) analyses, respectively.

For ambiviruses analysis, a multiple sequence alignment of RNA-dependent RNA polymerase (RdRp) proteins was generated with MAFFT v7.310 (113) using the—localpair --maxiterate 1,000 parameters, followed by manual refinement. The alignment retained positions with <50% gap content across sequences to prioritize conserved regions, enabling the incorporation of partial RdRp sequences despite their gap contributions. Phylogenetic reconstruction employed the LG + G4+I substitution model, selected as optimal via model testing. Resulting trees were visualized using the Interactive Tree of Life (iTOL) platform (67). To evaluate taxonomic classification, pairwise identity comparisons were conducted with the Sequence Demarcation Tool (68). SDT-derived pairwise identity plots and distance matrices were cross-referenced with International Committee on Taxonomy of Viruses (ICTV) species demarcation thresholds (69) to determine whether newly identified viroid sequences conformed to established species boundaries or represented novel taxa.

### *In vitro* self-cleavage validation

For confirmation of self-cleavage activity in the twister ribozyme containing ex-circRNAs, DNA templates for single-stranded RNA synthesis were obtained by PCR using gene-specific primers (Table S5) that contained the T7 polymerase recognition sequence at their 5' end. After ethanol precipitation, the transcript was dissolved in H2O treated with DEPC. The purified products were incubated at 80°C for 1 min and cooled to room temperature (25°C) in the self-cleavage buffer (30 mM HEPES, 100 mM NaCl, 20 mM MgCl_2_) and then extracted from 8% PAGE gel slices containing 8M urea and visualized by ethidium bromide staining. The *in vitro* self-cleaving transcripts were excised from the gels and eluted separately.

### CRISPR Spacer analysis for Obelisk plant origin assessment

Host-Obelisk associations were established by linking CRISPR-containing genomes to their corresponding Obelisk targets, and spacer conservation was assessed across related bacterial taxa. For this, CRISPR spacer sequences from bacterial and archaeal genomes in the Joint Genome Institute’s IMG/M database (117) were systematically searched against our Obelisk-like RNA using BLASTn (parameters: word size 11, E-value 1e–05) to test for evidence of prokaryotic targeting. The absence of significant spacer matches (no hits meeting ≥85% identity over ≥20 nucleotides) was documented across microbial genomes, supporting non-bacterial origins of these RNA elements.

### Quantification and statistical analysis

The abundance of the candidate viroid and viroid-like elements was expressed as a normalized result of the number of reads mapped to the candidate viroid. The reads in were aligned to the final viroid and viroid-like candidates with bowtie2 software (118), and the alignment parameter was "end-to-end–very-fast," and then, the number of reads aligned to each viroid-like sequence was calculated by SAMtools (119), and the coverage and depth were calculated by BEDtools (120). Mapped reads were counted for all aligned sequences. For each sequence, the total read count, coverage (percentage of the sequence spanned by reads), and depth (average reads per nucleotide) were determined. RPKM (Reads Per Kilobase per Million mapped reads) values were calculated by normalizing read counts to the total mapped reads in the library and the sequence length (in kilobases). Read abundance data, represented as RPKM values, was visualized using an R script.

## Data Availability

All data for this manuscript is available at Journal mSystems online. The sequence data of viroids and viroid-like RNAs is available at the public repository Zenodo (https://doi.org/10.5281/zenodo.17010633).

## SUPPLEMENTAL MATERIAL

Supplemental Figures msystems.01168-25-s0001.pdf Fig. S1 to S7.

Supplemental Tables msystems.01168-25-s0002.xlsx Tables S1 to S10.

ASM does not own the copyrights to Supplemental Material that may be linked to, or accessed through, an article. The authors have granted ASM a non-exclusive, world-wide license to publish the Supplemental Material files. Please contact the corresponding author directly for reuse.
