# Supplementary material for: Discovery of novel hammerhead, twister, and DVRz-associated circular RNAs in Vitaceae, Solanaceae, and Rosaceae
Source: mSystems. 2025 Sep 15;10(10):e01168-25. doi: 10.1128/msystems.01168-25 (PMC12542633; doi:10.1128/msystems.01168-25)
Supplement: Supplemental Figures — Fig. S1 to S7. [file msystems.01168-25-s0001.pdf]

Supplementary Figures

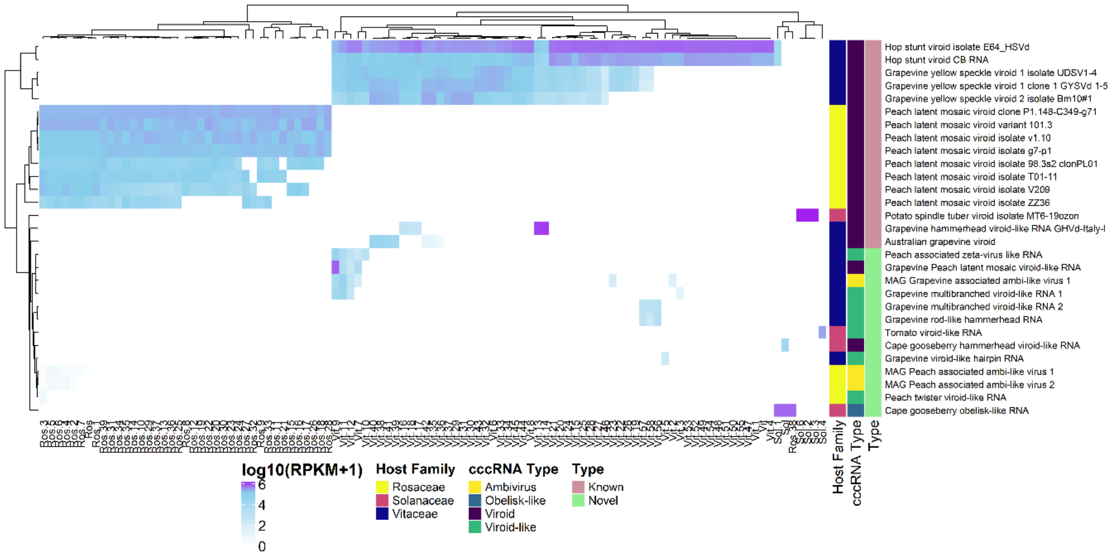

Supplementary Figure S1. The abundance and read mapping RPKM plot for all the detected ex-circRNAs in three families SRA. The rows (cccRNAs contigs) and columns (host families) are clustered based on their RPKM profiles. The legend at the bottom indicates the color coding for host families, cccRNA types, and the novel/known status, as well as the scale for the log10(RPKM+1) values.

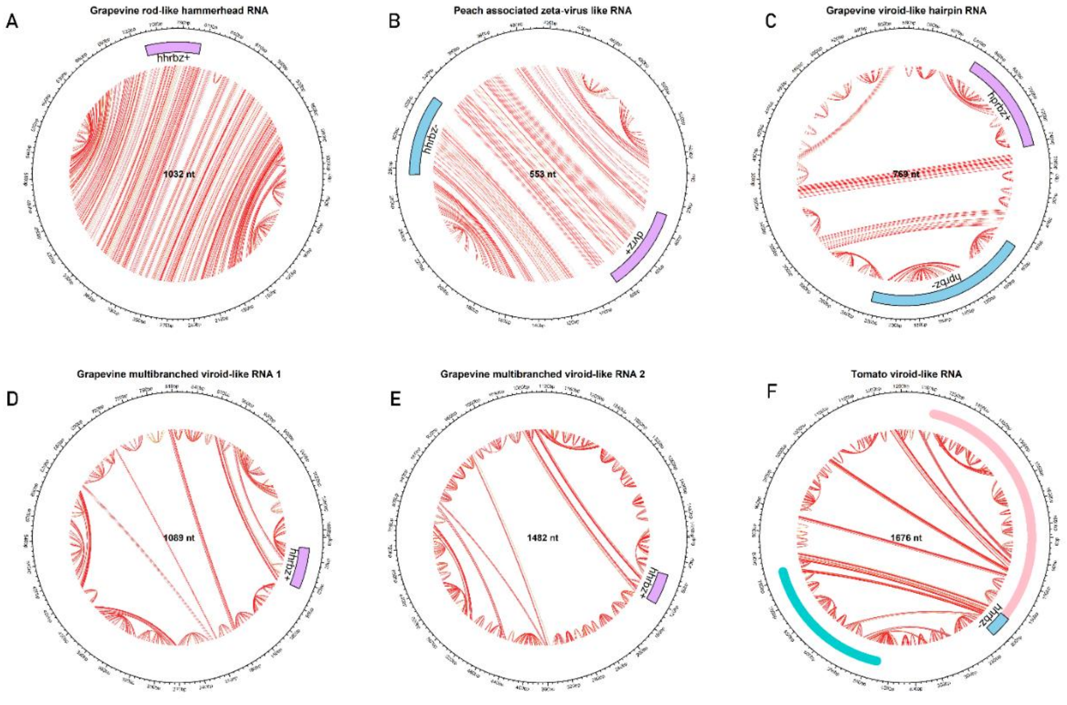

Figure S2. Circular genomic maps of plant viroid-like RNAs. A. *Grapevine rod-like hammerhead RNA (1032 nt)*. B. *Peach associated zeta-virus like RNA* (553 nt) (displaying terminal dvrz+ purple and hhrbz- blue, respectively). C. *Grapevine viroid-like hairpin RNA*. D. *Grapevine multibranched viroid-like RNA 1* (1089 nt) with hhrbz+ (purple) and distinctive internal branching pattern. E. *Grapevine multibranched viroid-like RNA 2* (1482 nt) showing complex secondary structure elements and hhrbz+. F. *Tomato viroid-like RNA* (1676 nt), displaying extensive secondary structure with hhrbz- and two predicted ORFs (green and red).

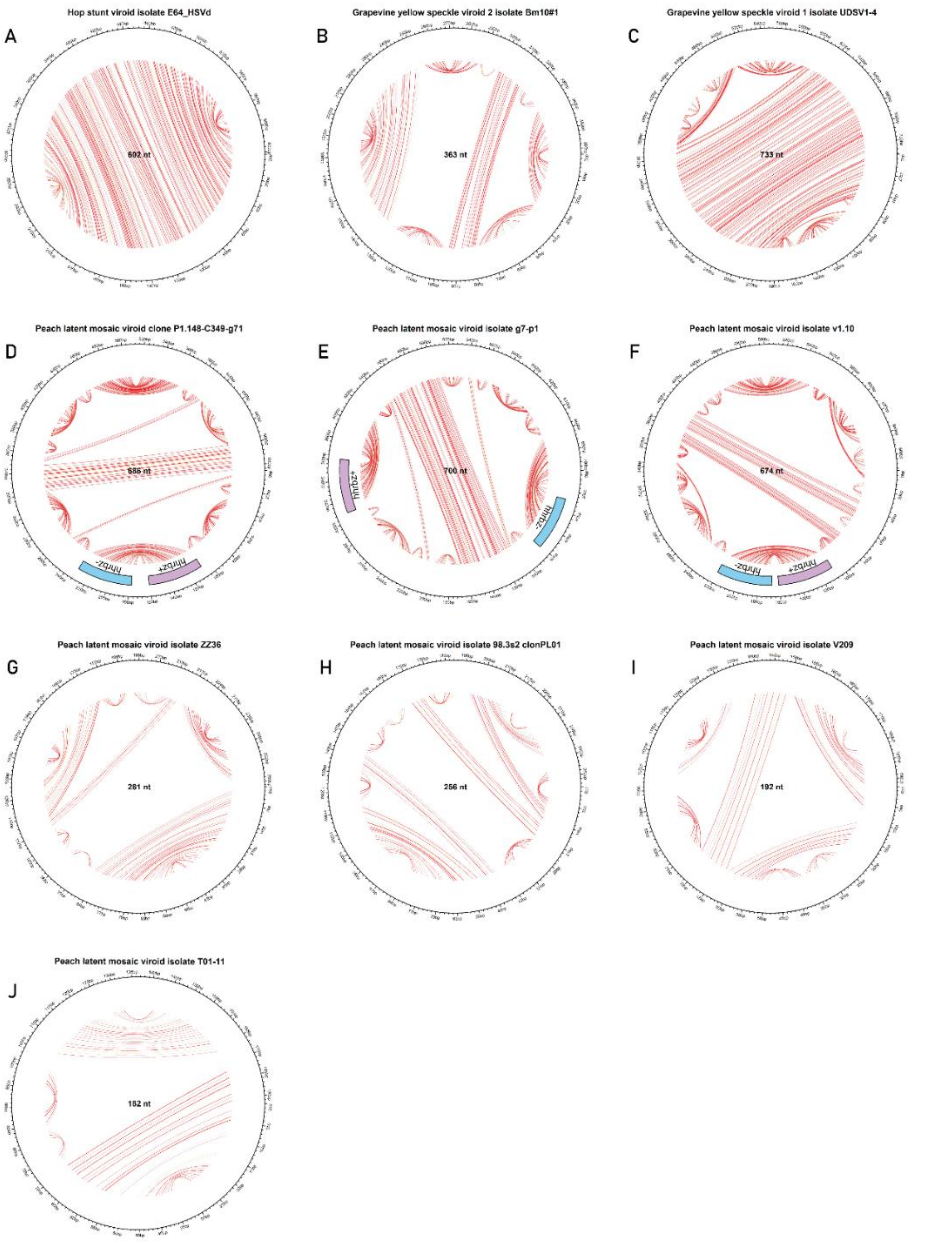

Figure S3. Circular genomic maps of viroids. A. *Hop stunt viroid* isolate E64_HSVd (602 nt) showing dense parallel base-pairing structure. B. *Grapevine yellow speckle viroid 2* isolate Bm10#1 (363 nt) with distinctive cruciform arrangement. C. *Grapevine yellow speckle viroid 1* isolate UDSV1-4 (733 nt) displaying extensive base-pairing interactions. D through J. *Peach latent mosaic viroid* isolates and variants: D. clone P1.148-C349-g71 (686 nt) with symmetric hhrbz (blue arcs); E. isolate g7-p1 (700 nt) showing hhrbz+ and hhrbz- domains (purple and blue arcs, respectively); F. isolate v1.10 (674 nt) featuring central paired regions and hhrbz- domains (blue and purple arcs); G. isolate ZZ36 (261 nt); (H) isolate 98.3x2 clonPL01 (256 nt); I-J. Small fragments. I. isolate V209 (192 nt); and J. isolate T01-11 (182 nt). Red lines represent predicted RNA secondary structure interactions (0-1).

RNABob program

Type I hammerhead ribozyme

```
h1 s1 h2 s2 h2' s3 h3 s4 h3' s5 h1'
h1 0:0  NNNNNN:NNNNNN
s1 0    CTGANGA
h2 0:0  GNNN:NNNC
s2 0    N[98]N
S3 0    GAA
h3 0:0  AN:NT
s4 0    N[28]N
s5 0    C
```

Type II hammerhead ribozyme

```
h1 s1 h2 s2 h2' s3 h3 s4 h3' s5 h1'
h1 0:0  NNNY:RNNN
s1 0    GAA*
h2 0:0  AN:NT
s2 0    N[98]N
S3 0    C
h3 0:1  NNNNNN:NNNNNN
s4 0    N[28]N
s5 0    CTGANGA
```

Type III hammerhead ribozyme

1. h1 s1 h2 s2 h2' s3 h3 s4 h3' s5 h1'

```
h1 0:0 NT:AN
s1 0    C
h2 0:0 NNNNN:NNNNN
s2 0    N[4998]N
S3 0    CTGANGA
h3 0:0 GNNN:NNNC
s4 0    N [28]N
s5 0    GAA
```

2. h1 s1 h2 s2 h2' s3 h3 s4 h3' s5 h1'

```
h1 0:0 ***NNN:NNN***
s1 0  TH
h2 0:0 ***NNNN:NNNN***
s2 0 NNNN[96]
s3 0 CTGANGA
h3 0:0 **NNNN:NNNN**
s4 0 NNNN[96]
s5 0 GAAA
```

HDV-Luptak

```
h1 r2 s1 r3 r4 s2 r5 s3 r4' r2' h1' r5' s4 s5 s6 r3'
h1 0:0 G:Y
r2 0:1 NNNNN*:*NNNNN TGCA
s1 0   NN[150]
r3 0:1 ***NNNNNN:NNNNNN***  TGCA
r4 0:0 NNN:NNN TGCA
s2 0   TY
r5 0:0 C:G TGCA
s3 0   HCG*Y
s4 0   N
s5 0   NNN[150]
s6 0   C*RA*
```

RNAmotif program

Type I hammerhead ribozyme

```
parms
 wc += gu;
descr
#typeI
  h5 (minlen=3, maxlen=12)
   ss (minlen=7, maxlen=8, seq="^cuganga")
  h5 (minlen=2, maxlen=12)
    ss (minlen=3, maxlen=100)
  h3
   ss (len=3, seq="gaa")
  h5 (minlen=3, maxlen=12, seq="^a")
    ss (minlen=3, maxlen=100)
  h3 (seq="u$")
    ss (len=1, seq="h")
  h3
ss (len=10)
```

Type II hammerhead ribozyme

```
parms
 wc += gu;
descr
h5 (minlen=2, maxlen=5)
  ss (len=3, seq="gaa")
  h5 (minlen=3, maxlen=12, seq="^a")
    ss (minlen=3, maxlen=100)
  h3 (seq="u$")
  ss (len=1, seq="h")
  h5 (minlen=3, maxlen=12)
    ss (minlen=3, maxlen=100)
  h3
  ss (minlen=7, maxlen=8, seq="^cuganga")
h3
ss (len=10)
```

Type III hammerhead ribozyme

```
parms
wc += gu;
descr
h5 (minlen=3, maxlen=12, seq="u$")
  ss (len=1, seq="h")
  h5 (minlen=3, maxlen=12)
    ss (minlen=3, maxlen=100)
  h3
   ss (minlen=7, maxlen=8, seq="^cuganga")
  h5 (minlen=2, maxlen=12)
    ss (minlen=3, maxlen=100)
  h3
   ss (len=3, seq="gaa")
  h3 (seq="^a")
 ss (len=10)
```

Figure S4. The Descriptor files used for detection of ribozymes by motif search tools i.e RNAbob and RNAmotif.

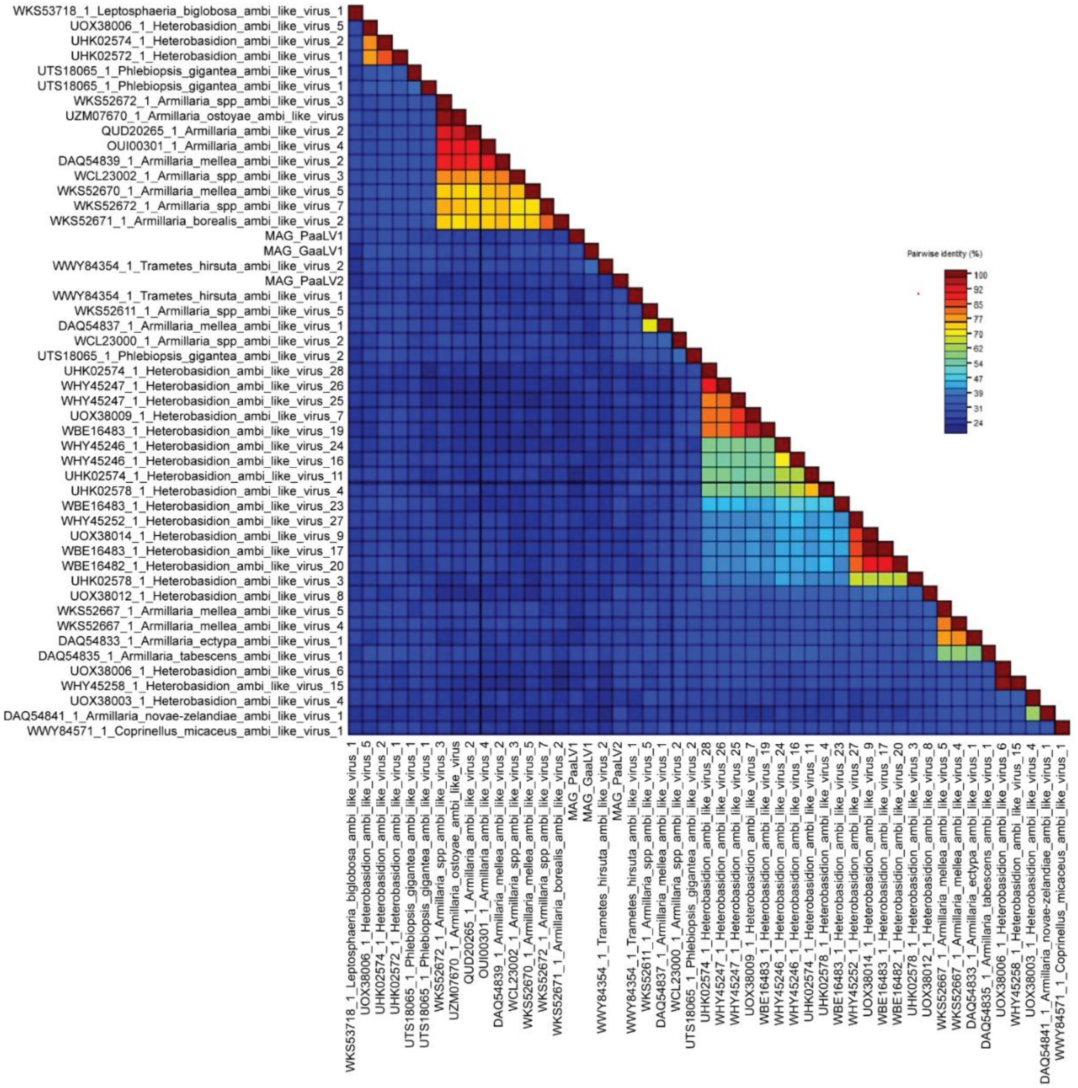

Figure S5. Pairwise nucleotide identity matrix of ambi-like viruses detected across diverse fungal hosts. The heatmap displays percent identity between 45 ambi-like viral isolates fungal genera. Viral isolates are labeled with their accession numbers and host species. Sequence identities range from 0% (dark blue) to 100% (dark red) (corresponding 0 to 1) as indicated in the color scale bar (right). Three notable isolates—MAG PaALV1, MAG PaALV2, and MAG GaALV1—form a distinct cluster showing high sequence conservation among themselves but low identity with other ambi-like viruses (predominantly dark blue regions). The divergence of the PaALV1/PaALV2/GaALV1 group from other mycoviruses suggests they represent a novel lineage within the ambi-like virus classification, potentially reflecting host adaptation or different evolutionary trajectories.

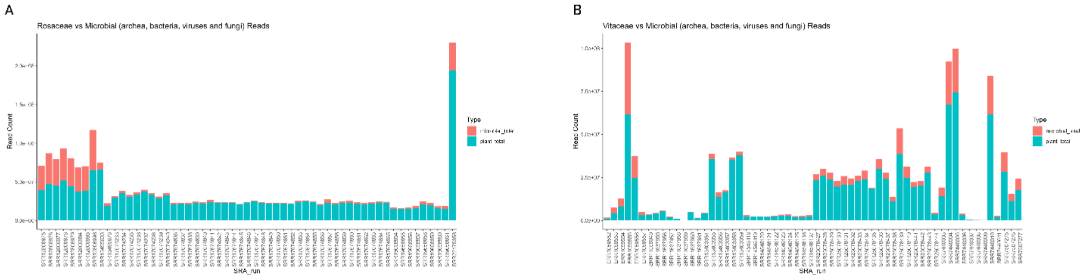

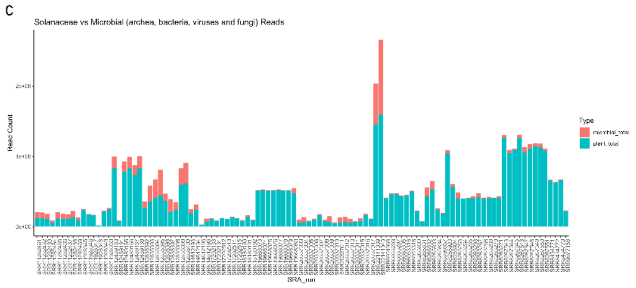

D

Statistic	Vitaceae	Rosaceae	Solanaceae
Total Samples	60	57	104
Average Plant Reads (%)	94.6	96.6	80.4
Average Microbial Reads (%)	5.4	3.4	19.6
Average Total Reads	12,174,429	23,239,477	36,434,051

Figure S6. Comparative analysis of sequencing read distribution between plant families and microbial communities. A. *Vitaceae* vs microbial reads, B. *Rosaceae* vs microbial reads, and C. *Solanaceae* vs microbial reads. Stacked bar charts show the proportion of plant-derived reads (red) versus total microbial reads including archaea, bacteria, viruses, and fungi (teal) across different SRA (Sequence Read Archive) samples. Read counts are displayed on a logarithmic scale to accommodate the wide range of sequencing depths across samples, with the Y-axis representing total read counts per sample. Microbial reads encompass all non-plant sequences including bacterial, archaeal, viral, and fungal origins as determined by Kraken2/Bracken (Table S7). SRA accession numbers are provided on the x-axis for reproducibility and data access, with sample ordering following numerical SRA identifier sequence. D. Comparative sequencing statistics across three plant families showing sample distribution, read composition, and sequencing depth. Data represents analysis of publicly available SRA datasets for *Vitaceae* (grape family), *Rosaceae* (rose family), and *Solanaceae* (nightshade family) SRA.

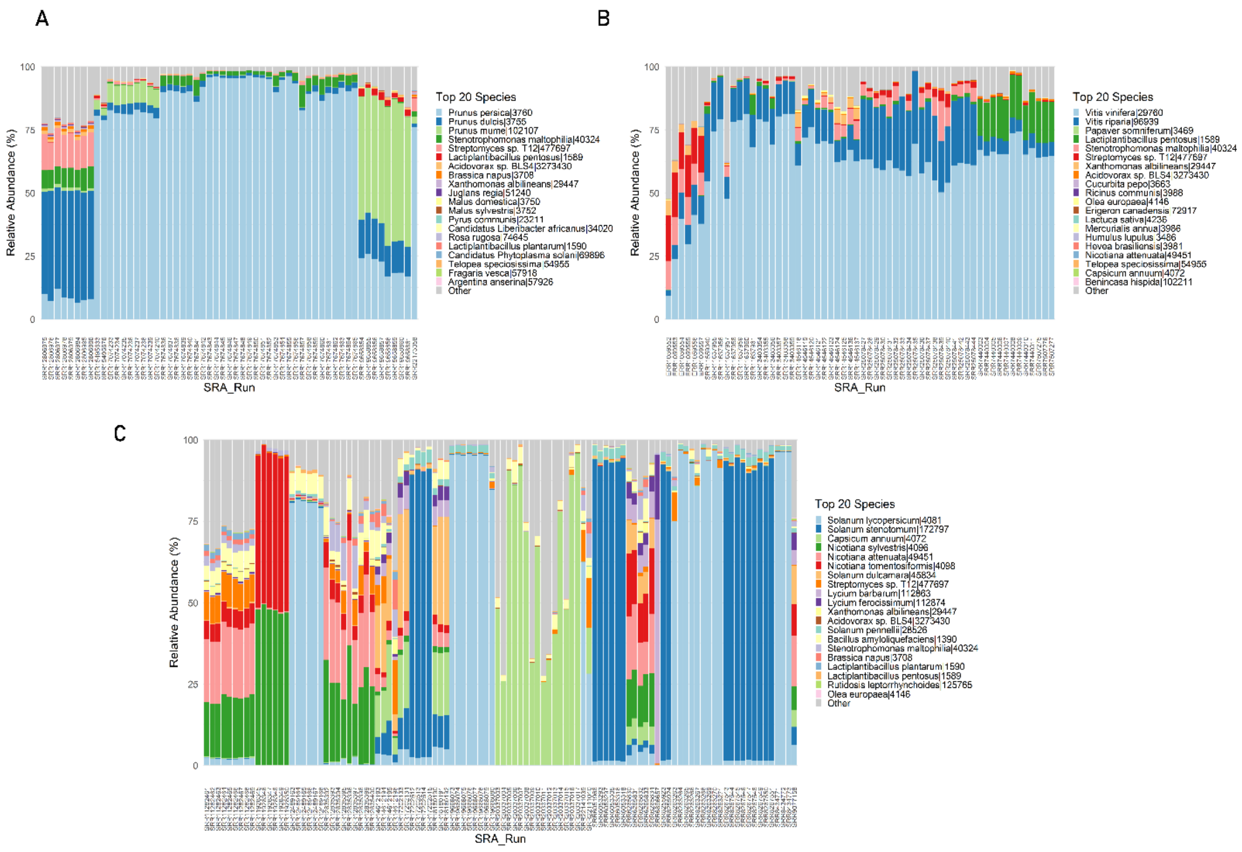

Figure S7. Taxonomic composition and relative abundance of the top 20 microbial species across SRA samples of three families. Stacked bar chart displays the percentage contribution of each species to the total microbial community, with samples ordered by SRA accession numbers. Species are color-coded according to the legend, with "Other" (gray) representing all remaining species not included in the top 20 most abundant taxa. The analysis reveals four main community types: A. *Prunus persica* appears as the most dominant species (light blue) (Table S8). B. *Vitis vinifera* as the dominant host plant (light blue) across most samples, with varying degrees of microbial colonization (Table S9). C. *Solanum lycopersicum* (light blue) emerges as the most abundant plant species, dominating numerous samples with up to 100% relative abundance, *Capsicum annuum* (pepper, yellow/orange) shows moderate presence as a secondary component in several samples, while *Nicotiana* species (*N. sylvestris* and *N. attenuata*, dark blue/purple) exhibit minimal representation across the dataset (Table S10).
